# Diterpenes with Abietane and Clerodane Skeletons from the Mexican *Salvia* Species as Potential Bioactive Compounds

**DOI:** 10.3390/molecules31183287

**Published:** 2026-09-16

**Authors:** Nancy Ortiz-Mendoza, Iván J. Bazany-Rodríguez, Martha J. Martínez-Gordillo, Francisco A. Basurto-Peña, Alejandro Dorazco-González, Itzi Fragoso-Martínez, María Eva González-Trujano, Emmanuel Martínez Ambriz, Marcos Soto-Hernández, Eva Aguirre-Hernández

**Affiliations:** 1 Laboratorio de Productos Naturales, Departamento de Ecología y Recursos Naturales, Facultad de Ciencias, Universidad Nacional Autónoma de México, Ciudad de México 04510, Mexico; nancy_om@ciencias.unam.mx; 2 Instituto de Química, Universidad Nacional Autónoma de México, Ciudad de México 04510, Mexicoadg@unam.mx (A.D.-G.); 3 Departamento de Biología Comparada, Herbario de la Facultad de Ciencias, Universidad Nacional Autónoma de México, Ciudad de México 04510, Mexico; 4 Jardín Botánico, Instituto de Biología, Universidad Nacional Autónoma de México, Ciudad de México 04510, Mexico; abasurto@ib.unam.mx; 5 Red de Biodiversidad y Sistemática, Instituto de Ecología A.C., Xalapa 91073, Mexico; itzi.fragoso@inecol.mx (I.F.-M.); emmanuel.martinez@inecol.mx (E.M.A.); 6 Laboratorio de Neurofarmacología de Productos Naturales, Dirección de Investigaciones Biomédicas en Salud Mental, Instituto Nacional de Psiquiatría Ramón de la Fuente Muñiz, Ciudad de México 14370, Mexico; evag@inprf.gob.mx; 7 Posgrado en Botánica, Colegio de Postgraduados, Campus Montecillo, Texcoco 56264, Mexico; msoto@colpos.mx

**Keywords:** abietanes, *Audibertia* subgenus, *Calosphace* subgenus, clerodanes, diterpenoids, Salvia

## Abstract

*Salvia* (Lamiaceae) is Mexico’s most diverse and widely distributed genus. It represents an important natural resource for the country, with approximately 310 species belonging to the subgenera *Calosphace*, *Audibertia*, and *Heterosphace*. Several of these species have been used in traditional Mexican medicine to treat various diseases. However, the main secondary metabolites as potential bioactive components have been poorly described. This review aims to identify the main chemical constituents of Mexican sages with abietane- and/or clerodane-type diterpene structures, list those with documented biological properties, and analyze the distribution of secondary metabolites within the genus from a phylogenetic perspective. The research identified at least 406 diterpene chemical components in Mexican salvias; of these, 128 were abietanes and 278 were clerodanes. Interestingly, some possess a broad spectrum of biological activities, including antidiabetic, antioxidant, antihypertensive, antimicrobial, antinociceptive, anti-inflammatory, and cytotoxic effects, among the most cited. Analysis of the distribution of abietanes and clerodanes in Mexican salvias showed a marked difference in their occurrence, since abietanes are mainly found in the *Audibertia* subgenus and clerodanes are more evident in the *Calosphace* subgenus. This review provides chemical and pharmacological information on diterpene metabolites in Mexican *Salvia* species, supporting the relevance of studying these species for future research on their potential bioactivity as a source of new therapeutic alternatives.

## 1. Introduction

Mexico is renowned for its great plant diversity and extensive medicinal flora, which serve as a source of ethnobotanical knowledge. The genus *Salvia* stands out for its species, which exhibit a broad spectrum of medicinal uses and contain secondary metabolites of pharmacological interest. Therefore, Mexico is considered one of the most important centers of diversification of the genus *Salvia*, with 75% of species endemic to the country [1,2]. These species belong to three distinct lineages of *Salvia* s.l. (subgenera *Calosphace* and *Audibertia* and the “Heterosphace” clade) The *Calosphace* subgenus of *Salvia* is the most represented, with at least 292 species. The species of this genus mainly comprise annual and perennial herbs, succulents, and shrubs, which are distributed in temperate forests, particularly coniferous, mesophytic, and oak forests, as well as in deciduous and subdeciduous forests and arid zones [3].

Species of the genus *Salvia* are used in traditional medicine to treat health conditions such as dysentery, diarrhea, gastritis, stomach pain, earache, headache, sore throat, cough, bronchitis, bile, fever, diabetes, epilepsy, nerves, insomnia, and anxiety, among others [4]. For metabolic diseases like diabetes, species such as *S. amarissima* Ortega, *S. lavanduloides* Kunth, and *S. leucantha* Cav. are used [4]. For gastrointestinal ailments including dysentery, diarrhea, stomach pain, inflammation, and colic, the most mentioned are *S. ballotiflora* Benth., *S. cinnabarina* M. Martens & Galeotti, *S. coccinea* Buc’hoz ex Etl., *S. elegans* Vahl, *S. hispanica* L., *S. lavanduloides* Kunth, *S. mexicana* L., and *S. polystachia* Cav. (Syn. *S. filipes*) [4,5,6,7]. In the case of central nervous system (CNS) disorders, *S. divinorum* Epling & Játiva, *S. elegans*, *S. fulgens* Cav., *S. leucantha*, *S. microphylla* Kunth, and *S. polystachia* (Syn. *S. filipes*) have been reported for their effects as tranquilizers to treat insomnia and epilepsy [4,6,8]. Interestingly, the main compounds isolated from salvias and identified as bioactive secondary metabolites are terpenic (monoterpenes, sesquiterpenes, diterpenes, and triterpenes) and phenolic in nature [4]. Among these, the abundance of diterpenes with abietane- and clerodane-type chemical structures stands out [4,6,9,10,11,12]. These have been shown to have antioxidant, antifeedant, antibacterial, anti-inflammatory, and cytotoxic properties, among others [4]. This review includes a list of endemic species and secondary metabolites, particularly diterpenic metabolites and those with biological activity, characterized by their chemical structures and abundance in these species. We also analyze their distribution within the genus’ phylogeny to understand the possible evolutionary pathway of diterpenes in Neotropical salvias. For this purpose, we performed an ancestral state reconstruction analysis using presence–absence data collected from various sources.

## 2. Materials and Methods

Although two molecular studies analyzed the relationships among the species of the subgenus *Calosphace* [13,14], there is still no formal proposal for an infrageneric classification since a greater number of markers and terminals need to be explored to avoid instability; for this reason, the Epling classification (1939) is used in this work. In the case of *Salvia dugesii* Fernald, *S. filipes* Benth. and *S. languidula* Epling, it is important to mention that they are currently considered synonyms of *S. melissodora* Lag., *S. polystachia* Cav., and *S. albiflora* M. Martens & Galeotti, respectively.

**Figure 1:**
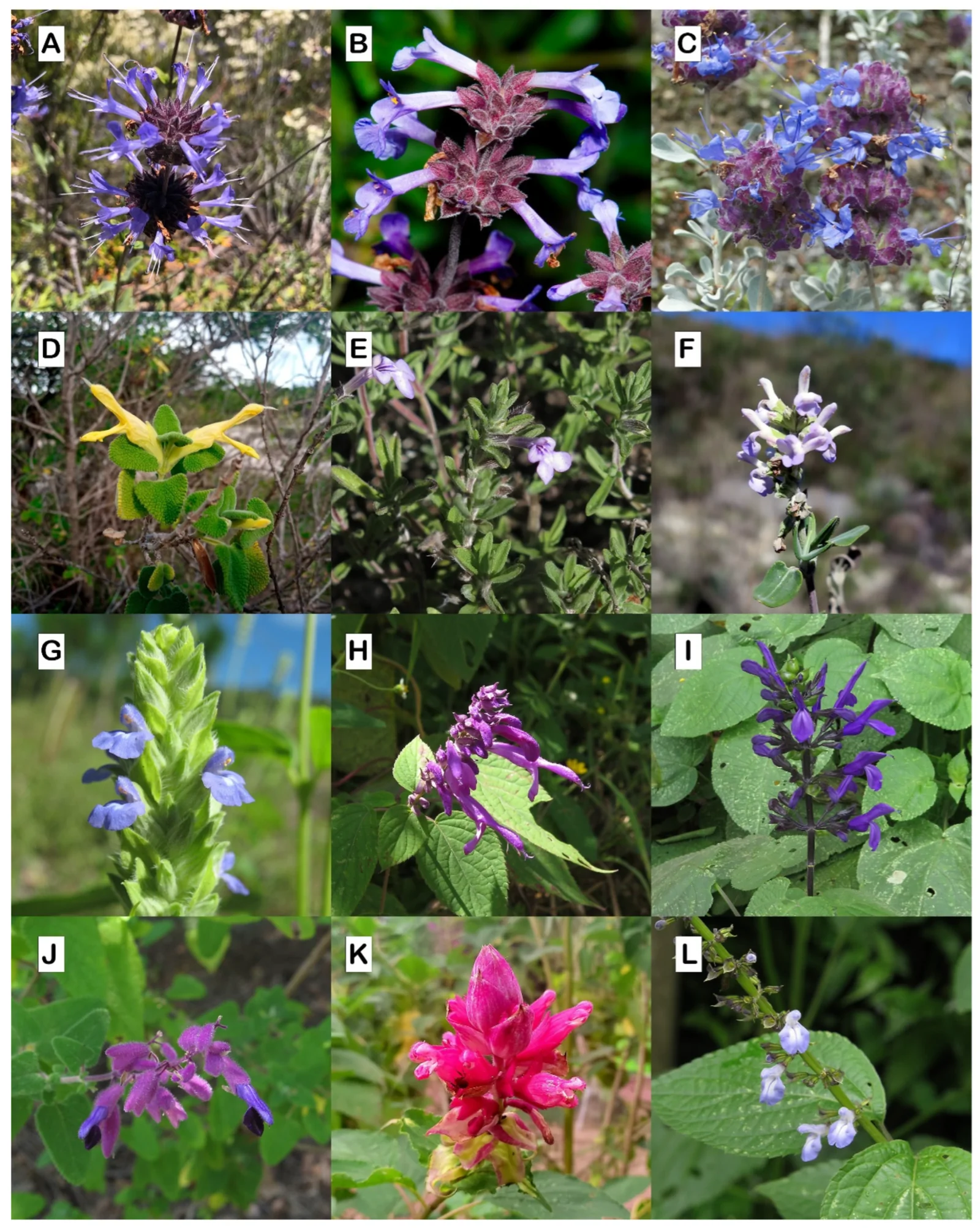
Mexican species of *Salvia*, subgenus *Calosphace* and *Audibertia* (**A**). *S. clevelandii*; (**B**). *S. munzii*; (**C**). *S. pachyphylla*; (**D**). *S. aspera*; (**E**). *S. axillaris*; (**F**). *S. candicans*; (**G**). *S. hispanica*; (**H**). *S. purpurea*; (**I**). *S. recurva*; (**J**). *S. semiatrata*; (**K**). *S. wagneriana*; (**L**). *S. xalapensis*. Photo credits: C. Jackson (**A**); A. Rockefeller (**B**); M. Marin (**D**); I. Fragoso (**C**,**E**,**F**,**H**–**J**,**L**); M. Martínez (**G**); E. Martínez (**K**).

We searched for characteristic metabolites of Mexican salvias by exploring the Scopus database and the UNAM thesis repository. The review covered the period from 1986 to 2025. Each binomial name of the 310 Mexican sage species listed in Supplementary Material Table S1 was entered into the SCOPUS search engine using the following search term: “Genus + species,” with the search fields set to “article title + abstract + keywords.” From the publications listed for each species, only those dealing with the structural elucidation of abietane- and/or clerodane-type compounds were selected. A total of 152 articles were selected. The data extracted from each manuscript included the compound name, chemical classification (abietane and/or clerodane), and species of origin. The information is organized in Supplementary Material Tables S1 and S2, and the chemical structures were drawn using ChemDraw 8.0 software. To uncover phylogenetic patterns in the distribution of diterpenoids within *Salvia* subgenus *Calosphace*, we constructed presence–absence matrices for abietanes and clerodanes based on literature reports. However, it is important to note that the isolation of these diterpenes depends heavily on the extraction techniques employed and the minimum concentration threshold required for detection. Consequently, the apparent absence of a given diterpene in the matrix does not rule out its actual presence in the plant; rather, it may reflect concentrations too low to be isolated using standard methodologies. The matrices included 88 taxa: 65 species from subgenus *Calosphace* and 23 representative species of other clades of *Salvia* s.l. (i.e., *Audibertia*, Glutinaria, “Heterosphace”, Rosmarinus, Salvia, *Salvia aegyptiaca*, and Sclarea) (Figure 1). The dated phylogeny of *Salvia* s.l. from [15] was used as a phylogenetic framework to reconstruct metabolite evolution in Neotropical sages.

We compared and matched the tips of the phylogenetic tree with the species included in the data matrices; the tips belonging to species that lacked data were pruned using the “drop.tip” function in the ape package in R [16,17]. To reconstruct ancestral metabolite states, we tested two different evolutionary models: one in which all states had the same transition rate (“ER”—equal rates model) and one in which each state had its own transition rate (“ARD”—all rates different model). We tested both models for the abietane and clerodane data matrices and compared their likelihood values to select the model that best fit the data. We then reconstructed the evolutionary history of each character using the maximum likelihood method with the chosen model and mapped the results on the tree using the phytools package version 2.5-2 [18].

## 3. Results

### 3.1. Chemical Compounds Found and Isolated from Mexican Salvia Species

Researchers have isolated and characterized approximately 500 compounds from native Mexican species of the *Salvia* genus. The most notable group of metabolites is diterpenes, particularly those with abietane- and clerodane-type skeletons [4,19]. This review presents 128 abietanes (**1**–**128**) and 278 clerodanes (**129**–**406**). The chemical names of the molecules, the species from which they were isolated, and the verification references are indicated in Supplementary Material Table S2.

### 3.2. Diterpenoids

Diterpenoids have their biosynthetic origin in the mevalonic acid pathway. Their formation begins with the condensation of farnesyl pyrophosphate, a C15 unit, and isopentenyl pyrophosphate to obtain geranylgeranyl pyrophosphate. From this latter structure, the synthesis of a wide range of diterpenes is possible through cyclization reactions. From a taxonomic point of view, some of these compounds, such as gibberellins (phytohormones), are common in the Plantae kingdom. In contrast, others are specialized in small systematic groups such as families and genera [20]. The genus *Salvia* is distributed in Mexico, where diterpenoids are the most numerous secondary metabolites. Based on their chemical structures, these are mainly classified into four subgroups, abietanes, clerodanes, labdanes, and pimaranes, with the first two being the most abundant and diverse so far [4].

### 3.3. Abietanes and Clerodanes

**Figure 2:**
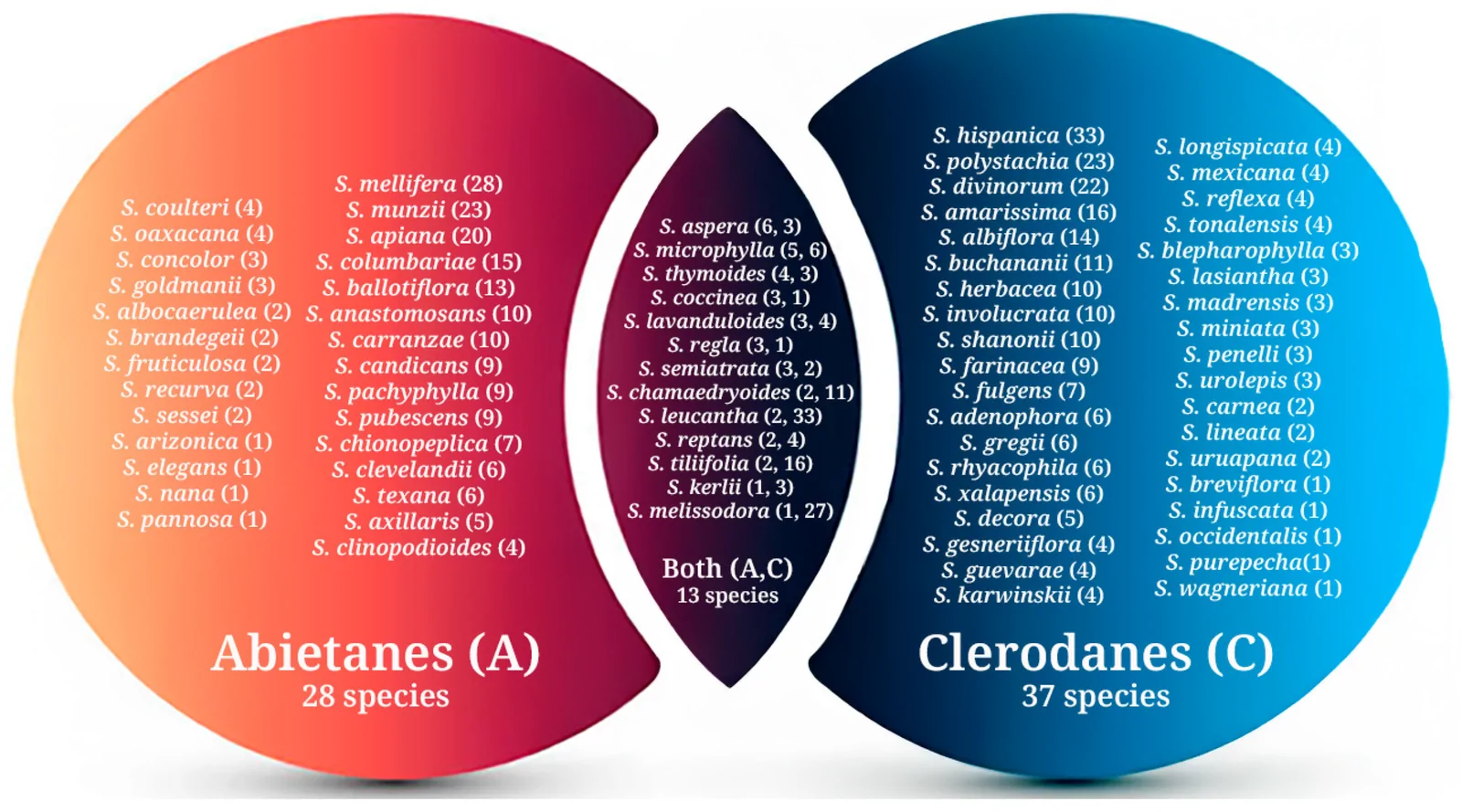
Abietanes and clerodanes isolated and identified in Mexican *Salvia*. Abietanes (A), clerodanes (C), and both (A, C).

Chemical structures belonging to the abietane and clerodane groups have been identified in 13 species of *Salvia* subgenus *Calosphace*, with a greater diversity of the latter compounds in *S. leucantha* (2 abietanes and 33 clerodanes), *S. melissodora* (Syn. *S. dugesii*) (**1**, **27**), and *S. tiliifolia* (**2**, **16**) (Figure 2) [21,22,23,24,25,26,27,28,29,30,31,32].

It is essential to mention that all the structures considered in this review fall within the classification of clerodanes and abietanes, or their derivatives. From a biosynthetic perspective, they likely originate from geranylgeranyl pyrophosphate, with many parallel pathways involved in producing the wide variety of abietane- and clerodane-type natural products found in *Salvia* [33,34].

### 3.4. Abietane Diterpenes from Salvia Species

**Figure 3:**
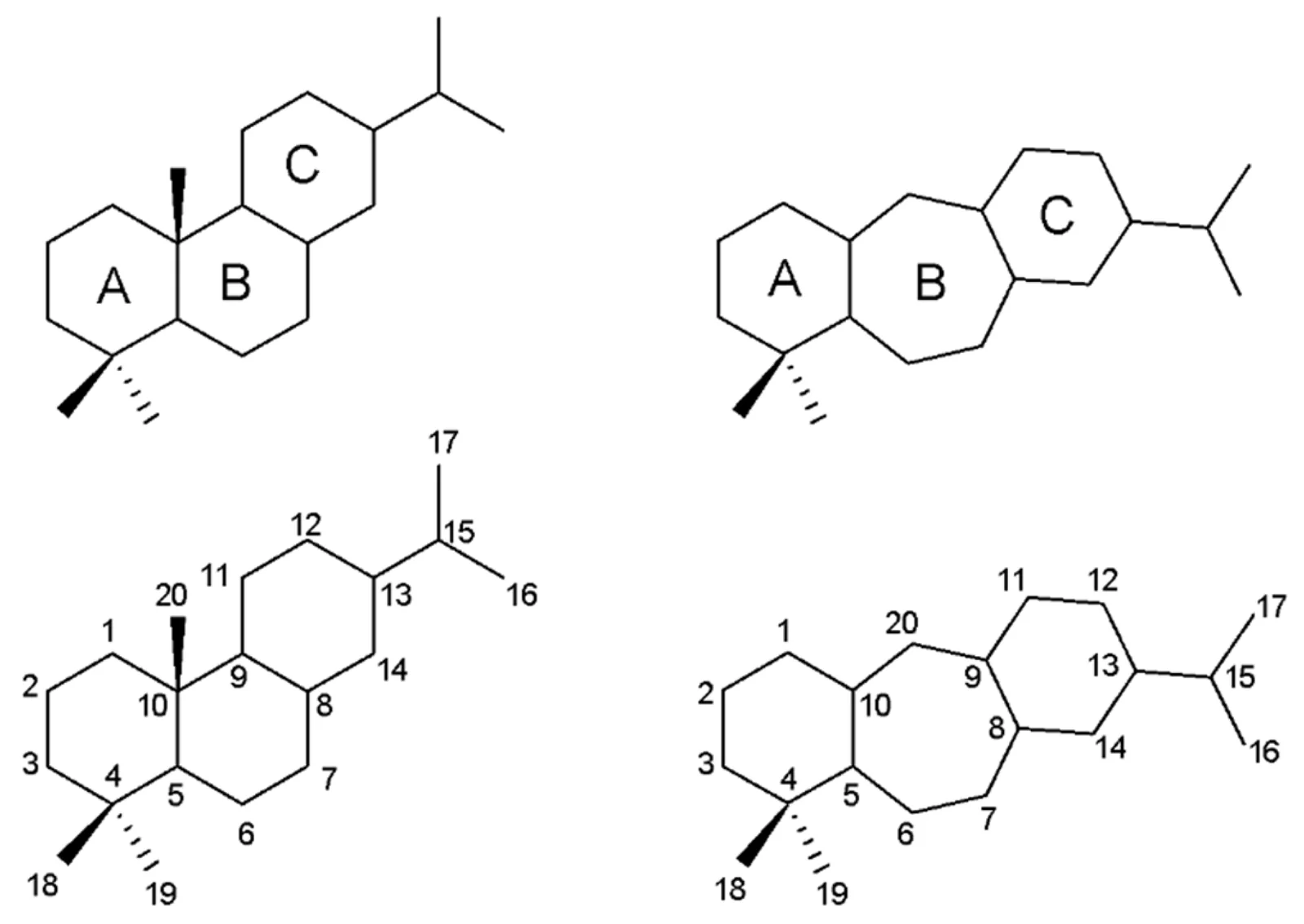
The skeleton of abietane-type structures and their icetexane-type sub-classification.

Abietanes are characterized by a carbon skeleton, as shown in Figure 3, with conjugated double bonds that exhibit characteristic UV spectra. These tricyclic compounds have different degrees of oxidation at the carbons positioned in the B- and C-rings. Depending on this, this group of metabolites is subclassified, as is the case with icetexane-type abietanes [35,36].

A total of 128 structures (**1**–**128**) have been isolated and identified from 41 species of Mexican salvias (Supplementary Material Table S1). Twenty-eight of these primarily synthesize abietanes, of which 20 belong to the subgenus *Calosphace*, seven to the subgenus *Audibertia*, and one to the subgenus *Heterosphace*. A large number of constituents have been identified in each of the species of the subgenus *Audibertia*, *S. mellifera* (28 abietanes), *S. munzii* (23), *S. apiana* (20), *S. columbariae* (15) and *S. pachyphylla* (9) [37,38,39,40,41,42,43,44,45,46]. On the other hand, species from the subgenus *Calosphace* that stand out include *S. anastomosans*, *S. carranzae*, *S. ballotiflora*, and *S. pubescens*, from which a significant number of structures have been identified (10, 10, 13, and 9, respectively) [10,47,48,49,50]. It is worth noting that of the 128 abietanes characterized so far, 83 (**1**–**83**) have been isolated from species of both the subgenera *Calosphace* and *Audibertia*, while 45 of these structures have been identified only in species of the subgenera *Audibertia* and *Heterosphace* (**84**–**128**). Abietanes are frequently found in the subgenera of *Salvia* found in the Old World, some of which are important for their biological properties, which have led to their frequent use in the *Pharmacopoeia* of this region; among the most important species are *S. officinalis*, *S. rosmarinus*, *S. miltiorrhyza*, and *S. sclarea* [51,52,53,54].

**Figure 4:**
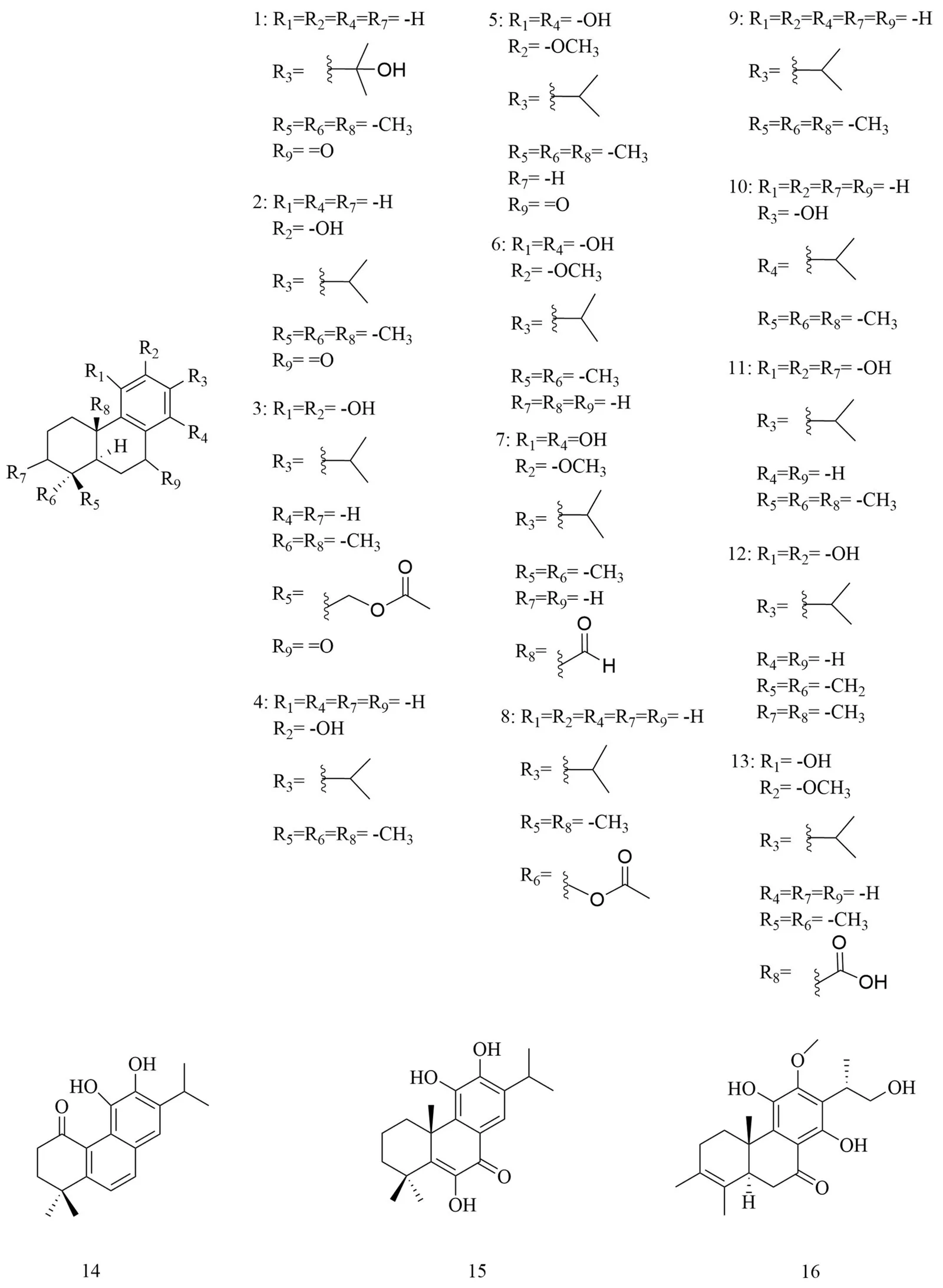
Abieta-8,11,13-trienes identified in Mexican salvias.

Only abietanes have been isolated from *S. axillaris*, the most basal species of *Calosphace* and phylogenetically closest to *Audibertia* species. This species and those belonging to *Audibertia* and *Heterosphace* grow in dry areas, particularly in xerophilous scrubland, with shallow soils and high-temperature variation throughout the year, which also generally applies to species in the sections *Tomentellae* (*S. anastomosans*, *S. ballotiflora*, *S. candicans*, *S. coulteri*, *S. fruticulosa*, and *S. goldmanii*) and *Conzatiana* (*S. oaxacana* and *S. aspera*) (Supplementary Material Table S1). According to their chemical characteristics, these 128 structures can be grouped into five subgroups:

**Abieta-8**,**11**,**13-trienes.** These structures are characterized by having an aromatic ring and different functional groups at C-3, 7, 11–14, and 18–20 (**1**–**16**) (Figure 4). Within this sub-classification, sugiol (**2**) and ferruginol (**4**) are the ones that have been identified in a greater number of species (Supplementary Material Table S2) [39,41,43,55,56,57,58,59,60,61]. These compounds are found in the three subgenera, *Audibertia*, *Calosphace*, and *Heterosphace*, in seven species of the first, 11 sections of the second, and one species of the third (*S. texana* (Scheele) Torr.), where the section *Tomentellae* stands out with four structures and *S. axillaris* Moc. et Sessé ex Benth. (Section *Axillares*), species where clerodanes have not been reported despite belonging to *Calosphace*.

**Figure 5:**
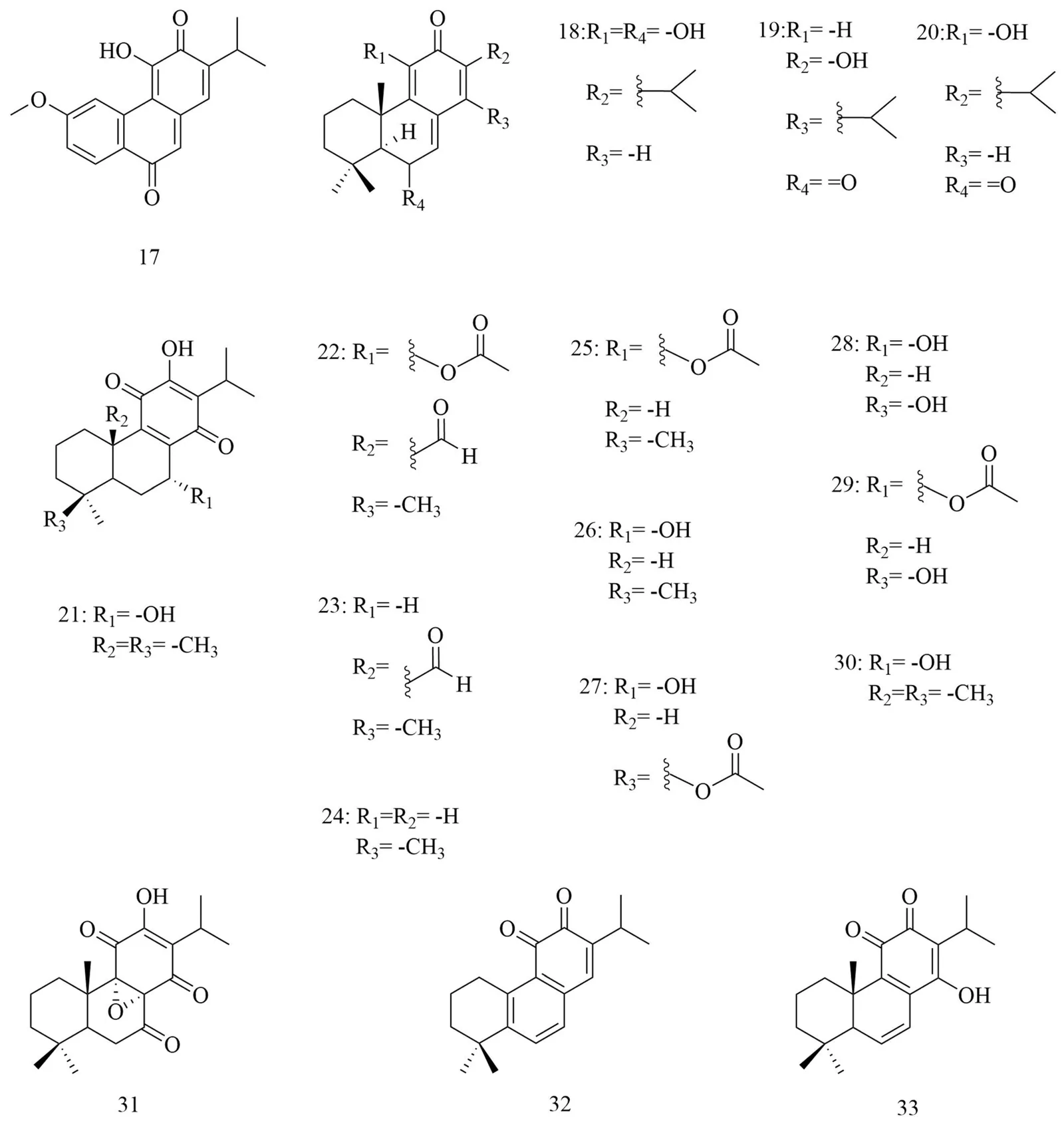
Abietanones identified in Mexican salvias.

**Abietanones.** This subgroup includes abietanes, characterized by having a single ketone group at C-12, or two at C-11 and C-12 or C-11 and C-14. Twenty-eight such structures have been isolated from Mexican salvias (**17**–**34**) (Figure 5). Among the abietanones found in species of the subgenus *Calosphace*, royleanone (**24**) and its derivatives (**25**–**29**, **31**, and **33**) stand out [39,43,48,62,63,64,65,66,67,68]. They have been recorded in both *Audibertia* and *Calosphace.* In the first subgenus, eight species have been isolated among those studied; in the second, 10 sections were represented, with the section *Tomentellae* standing out again with three structures, as well as *S. axillaris*, the most basal species of *Calosphace*.

**Figure 6:**
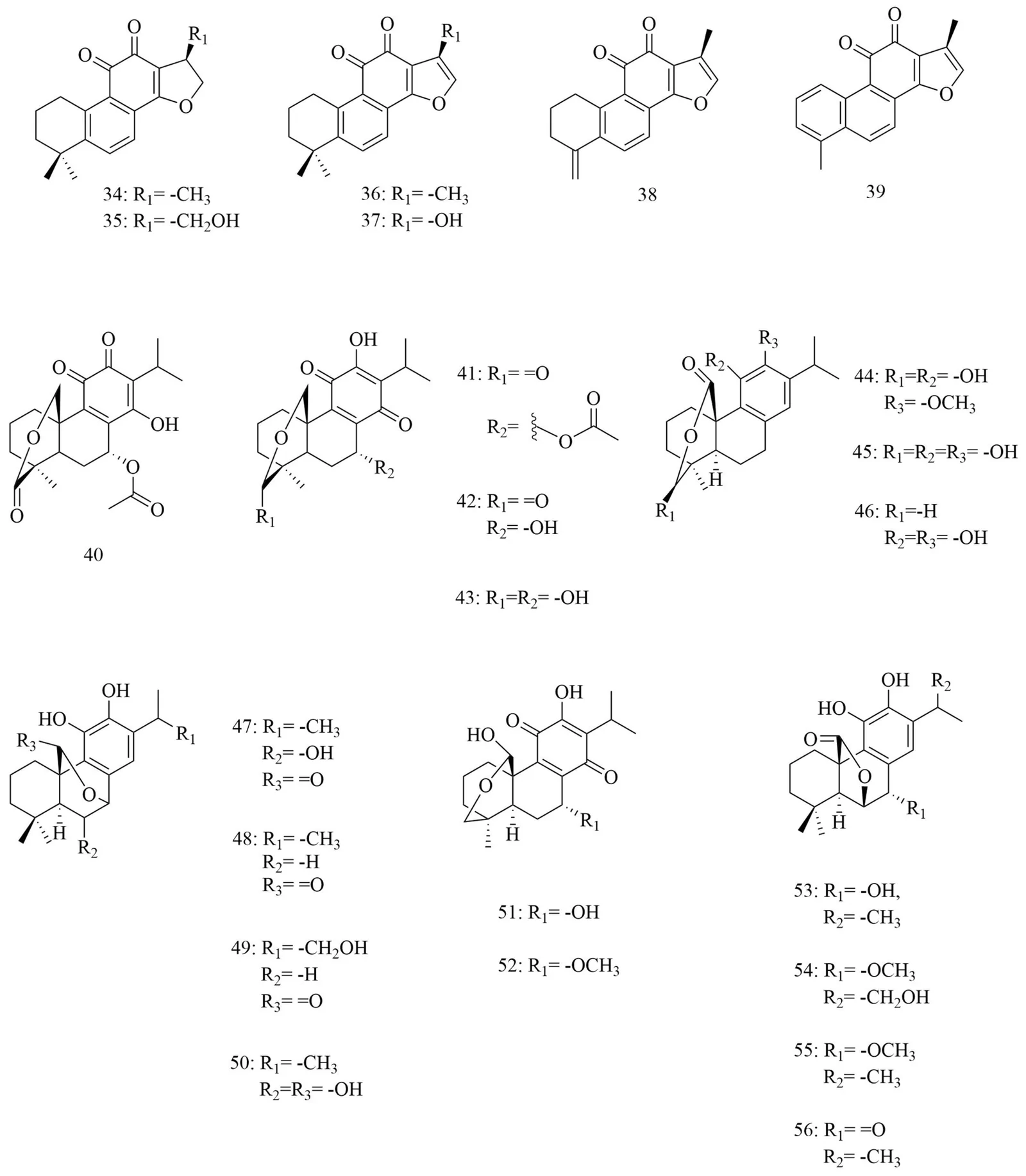
Epoxyabietanes identified in Mexican salvias.

**Epoxyabietanes**: This type of secondary metabolite is the most common to date in Mexican salvias (**34**–**56; 109**–**122**) (Figure 6). These structures are generally characterized by having an aromatic ring and functional groups such as ketone or hydroxy at C-11, C-12, C-14, and C-20, in addition to a 6,20-epoxy-, 7,20-epoxy, 14,17-epoxy-, or 19,20-epoxyabietanes substitution pattern. Carnosol (**48**) and rosmanol (**53**) are structures that have been isolated from a greater number of species of the subgenus *Calosphace* (Supplementary Material Table S2) [25,39,40,45,46,69,70,71,72,73,74,75]. The eight species of the subgenus *Audibertia* present structures of this type and seven sections of *Calosphace*, where *Tomentellae* stands out with four species that have these compounds.

**Figure 7:**
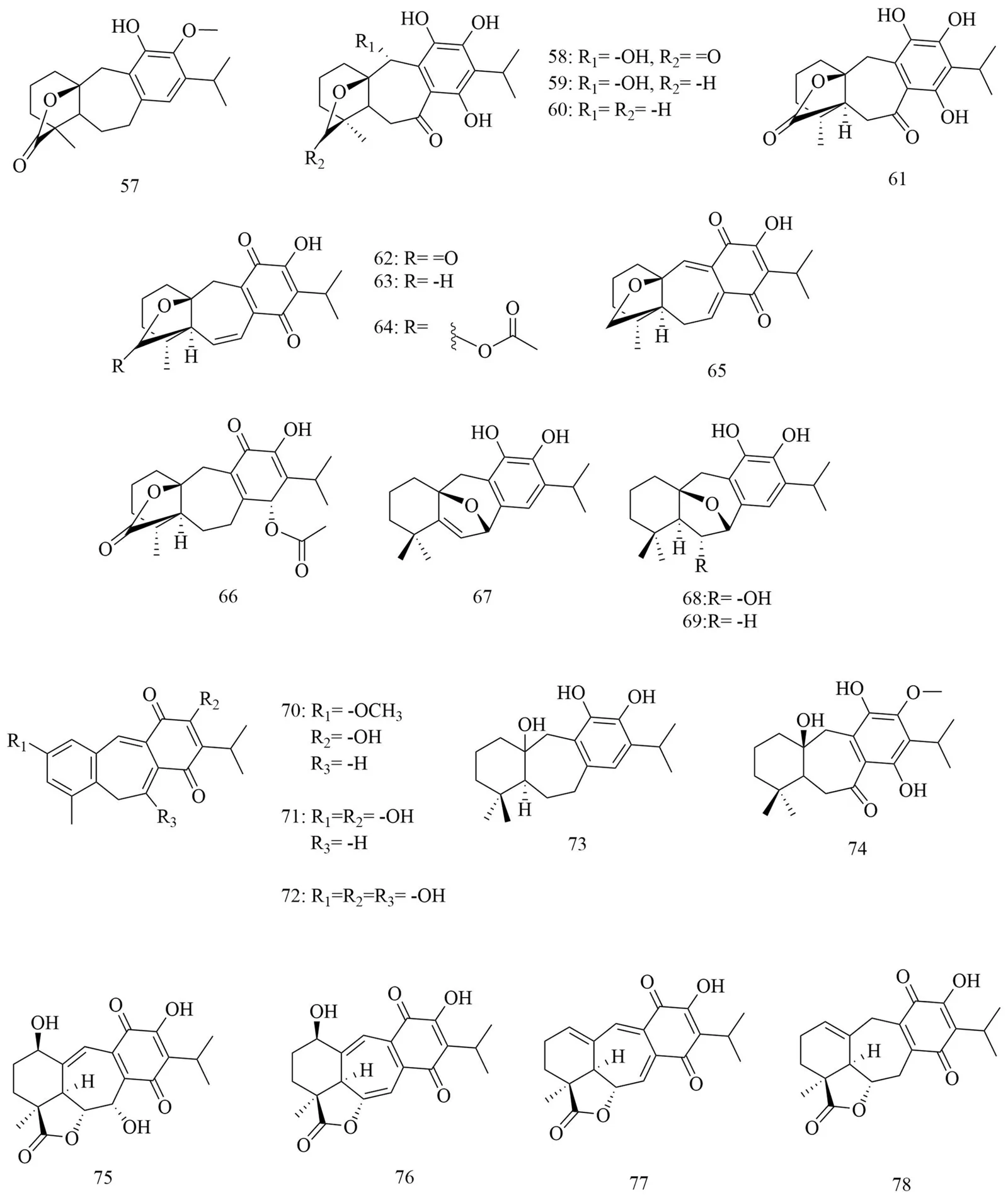
Icetexanes identified in Mexican salvias.

**Icetexanes**. These compounds arise biosynthetically from a rearrangement of the most common abietane and chemically belong to the 9 (10 -> 20) abietane skeleton with a 6/7/6 ring system (Figure 7) [19]. A total of 22 icetexanes (**57**–**78**) have been isolated from Mexican salvias, most of them from species of the subgenus *Calosphace*. Common arrangements include an epoxy group in the A or B ring (**57**–**69**) or the presence of a ɣ-lactone ring (**75**–**78**). These structures have been identified mainly in *S. anastomosans*, *S. ballotiflora*, *S. carranzae* and *S. candicans* [10,11,49,58,59,76,77,78,79,80]. The structures found in the most species are icetexone (**59**) and demethylsalvicanol (**70**) ([10,39,49,58,59,77,81]. In the subgenus *Audibertia*, they have been isolated from four species. In *Calosphace*, they have been reported in five sections, where *Tomentellae* stands out again, as they were found in the six species studied.

**Figure 8:**
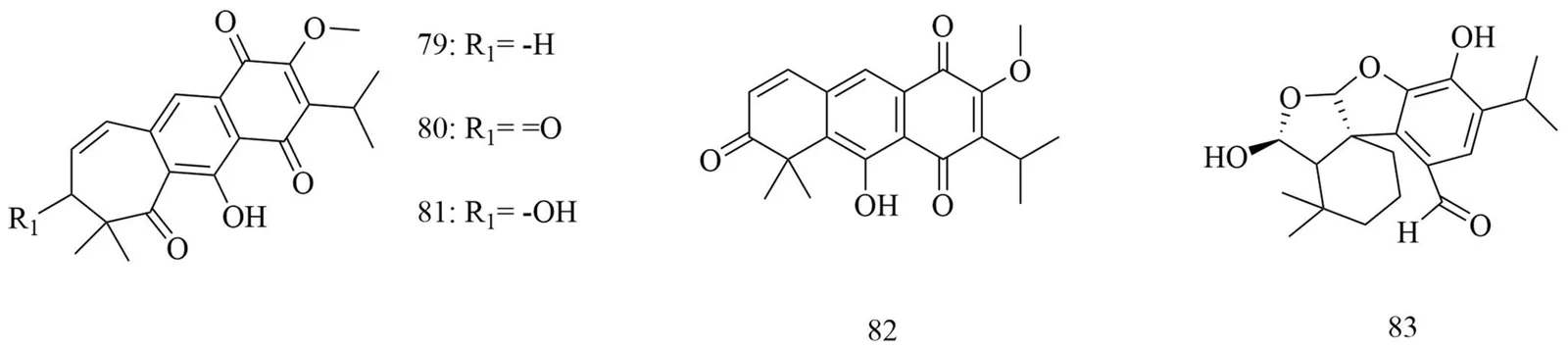
Other abietanes identified in Mexican salvias.

**Other abietanes***:* A total of nine compounds (**79**–**83**, **125**–**128**) (Figure 8) belong to this subgroup and, because of their chemical characteristics, are not included in any of those mentioned above. Of the structures, **79**–**83** isolated from species of the subgenus *Calosphace* are tilifolidione (**79**) and derivatives, identified in *S. thymoides*, *S. semiatrata*, and *S. tiliifolia* [24,82], and cariocal from *S. anastomosans* Ramamoorthy, and *S. candicans* M. Martens et Galeotii, both from the section *Tomentellae*. From the subgenus *Audibertia*, salvicanaric acid is reported from *S. munzii* ([39], 2α-hydroxysalvicanaric acid from *S. apiana*, *S. clevelandii*, and *S. mellifera* [45,46,81], and 16-hydroxy-rosmadial from *S. mellifera* ([38]. From the subgenus Heterosphace, 2α-hydroxysalvicanaric acid was isolated from *S. texana* (Scheele) Torr. [60].

**Figure 9:**
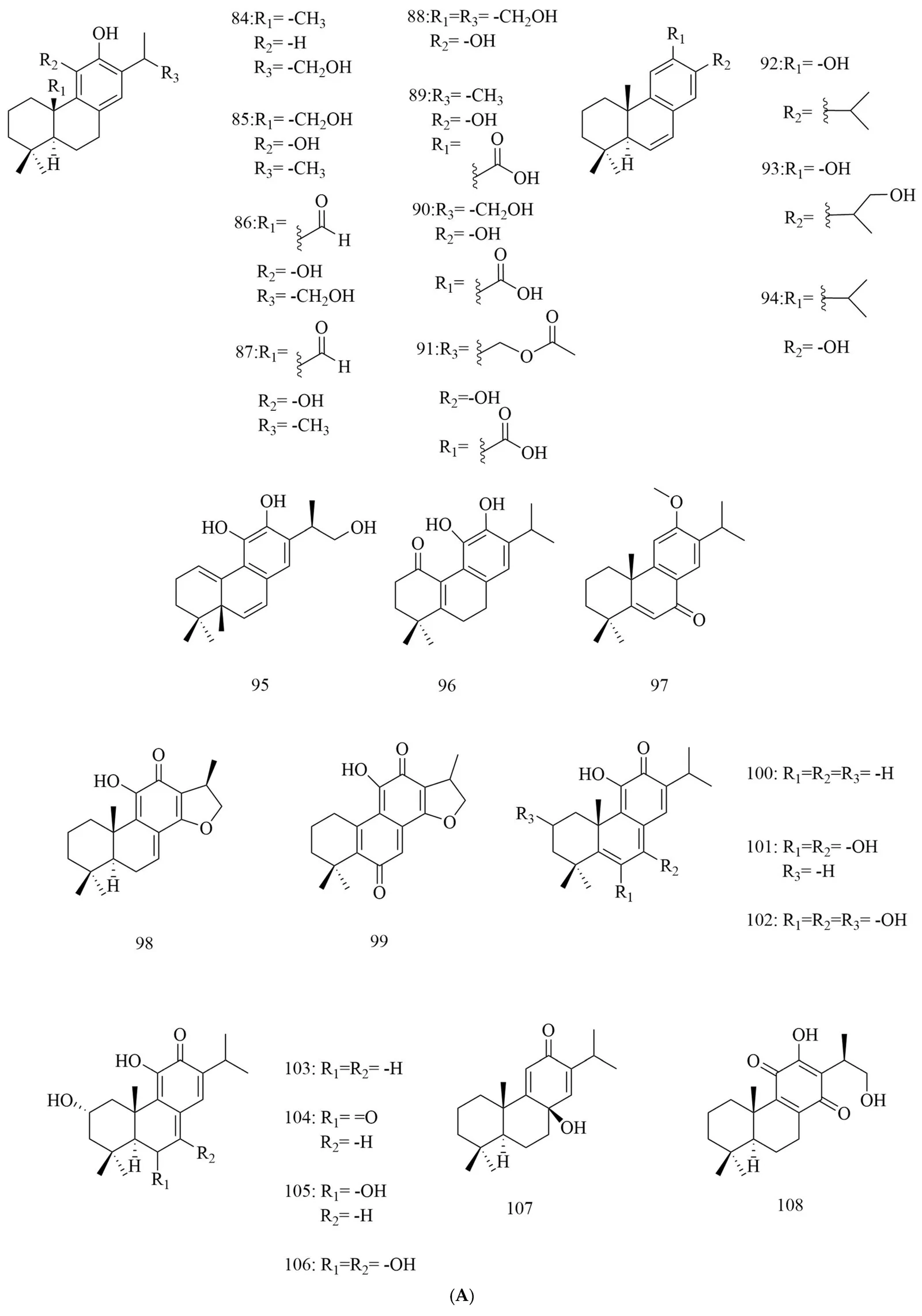

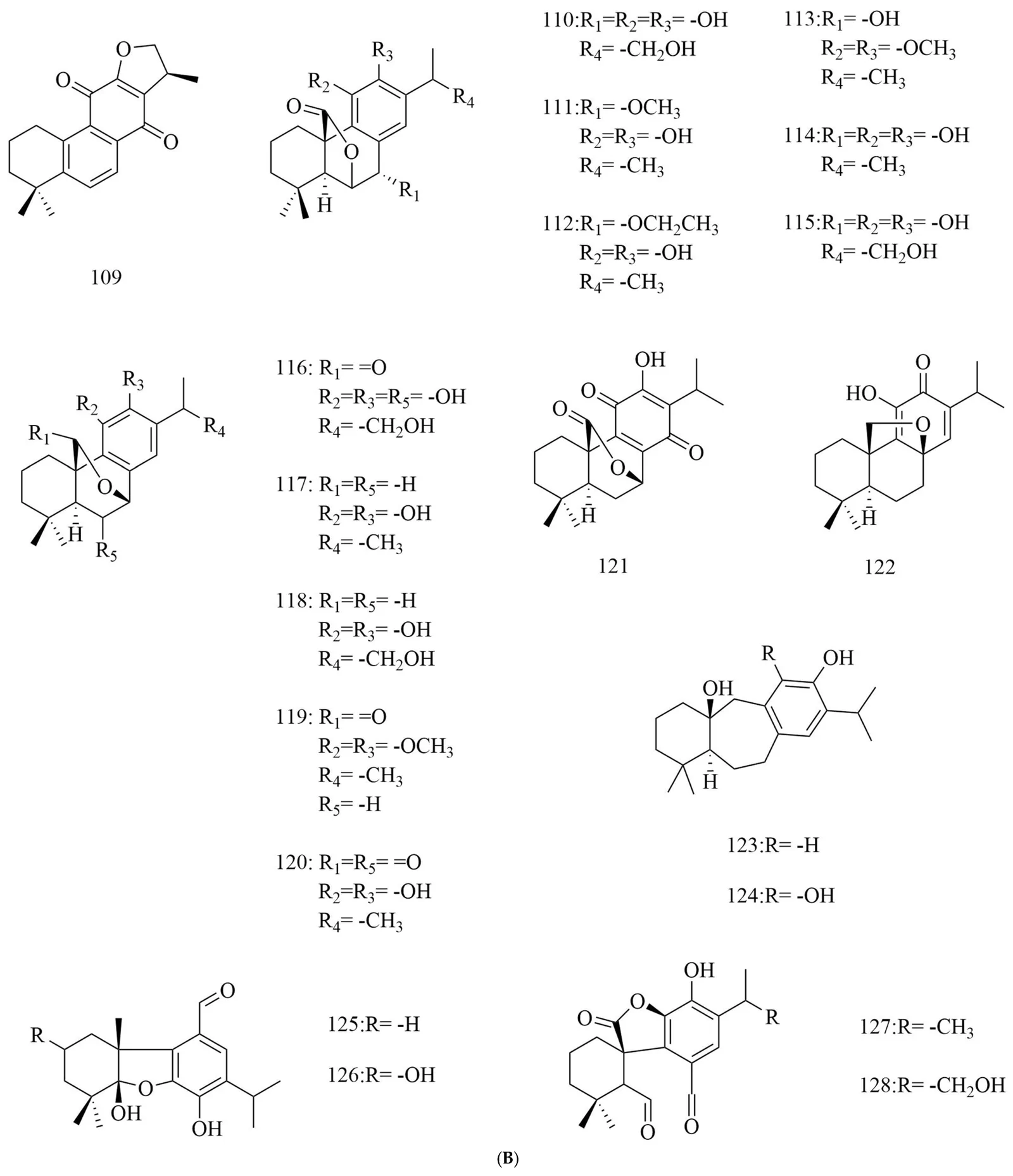
(**A**). Abietanes isolated from Mexican species of *Salvia* of the subgenus *Audibertia*. (**B**). Continued. Abietanes isolated from Mexican species of *Salvia* of the subgenus *Audibertia*.

***Abietanes in subgenus Audibertia and Heterosphace***. A total of 45 structures (**84**–**128**) have been isolated only from species belonging to the subgenus *Audibertia* (Figure 9). The species from which these compounds have been characterized are *S. apiana*, *S. chionopeplica*, *S. clevelandii*, *S. columbariae*, *S. mellifera*, *S. munzii*, *S. pachyphylla*, and *S. texana*. Compounds **84**–**128** belong to one of the categories described above and are predominantly composed of structures with abieta-8,11,13-triene (**84**–**97**)- and epoxyabietane (**110**–**122**)-type skeletons. Among these are compounds derived from ferruginol, carnosic acid, rosmanol, carnosol, and rosmadial are found, metabolites that are found in various *Salvia* species worldwide [4,25,38,39,60,70,72,83,84,85].

### 3.5. Clerodanes Diterpenes from Salvia Species

**Figure 10:**
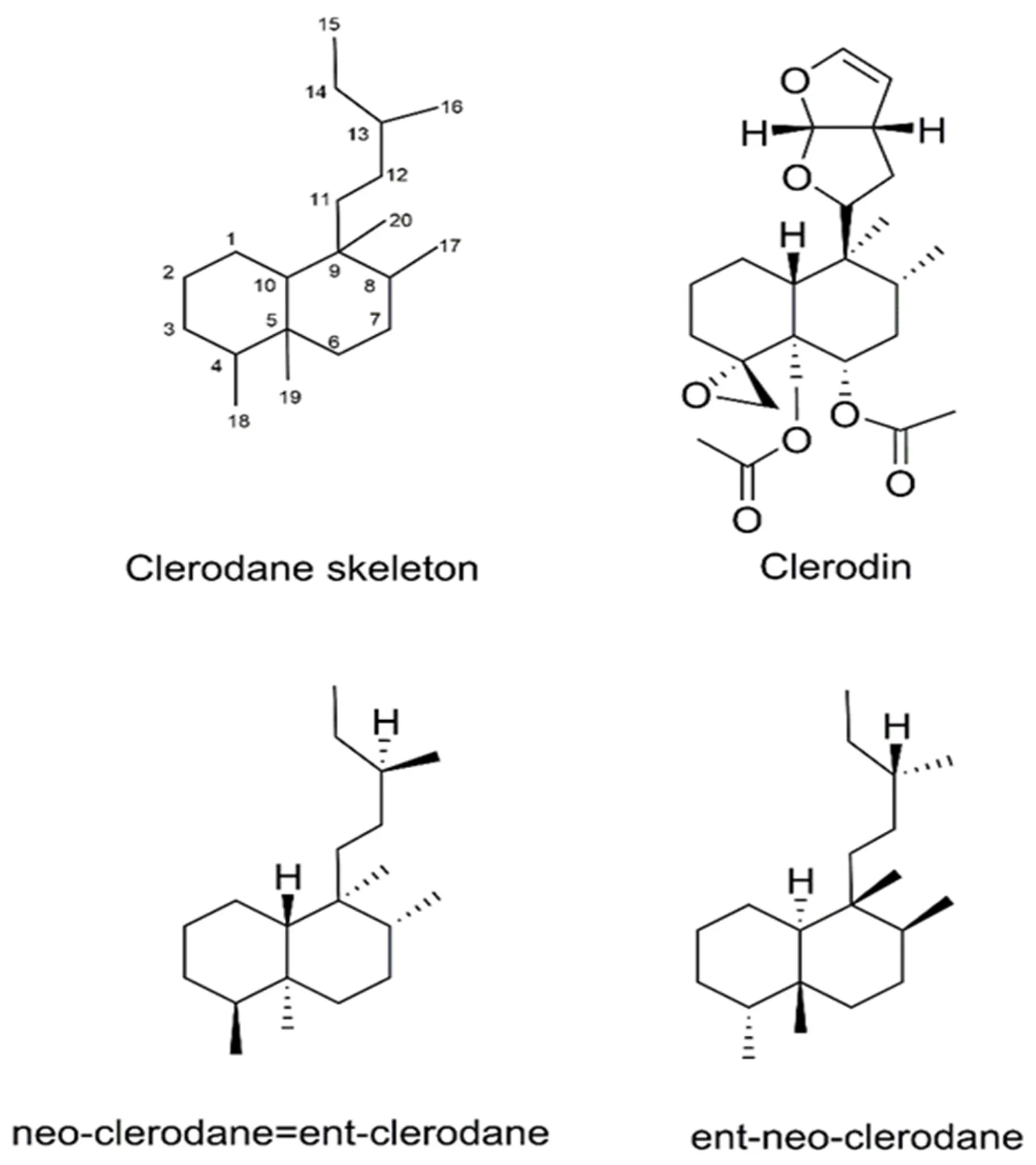
The basic structure and stereochemistry of clerodane diterpenes.

Clerodane diterpenes are bicyclic. The basic skeleton is divided into two fragments: a fused-ring decalin moiety (C-1-C-10) and a six-carbon side chain at C-9 (C-11-C-16), with C-16 attached at C-13. The remaining four carbons (C-17-C-20) are attached at C-8, C-4, C-5, and C-9, respectively, on the decalin system (Figure 10). Approximately 25% of clerodanes have a 5:10 cis ring fusion; the remaining 75% have a 5:10 trans ring fusion. Clerodin is the first member of the clerodane series, and compounds with the same absolute stereochemistry are called neo-clerodanes, while entneo-clerodanes are enantiomeric to clerodin [33,86]. Clerodanes comprise the majority of molecules identified from Mexican salvias, all belonging to the subgenus *Calosphace*. A total of 278 clerodanes are known (**129**–**406**) and have been isolated from 53 *Salvia* species. *S. hispanica* and *S. divinorum* are the species with the highest number and diversity of such chemical structures, of which 33 and 22 compounds are known, respectively [8,87,88,89,90]. They are followed by *S. amarissima* (16), *S. polystachia* (15), *S. duggesii* (12), *S. buchananii* (11) and finally, *Salvia herbacea* and *S. shannonii* with 10 clerodanes identified in each (Supplementary Material Table S2) [12,91,92,93,94,95,96,97,98,99,100].

It is interesting to note that, although clerodanes are found in many families worldwide, the *Salvia* species that exhibit this type of diterpenes are found mainly in the Americas. The different clerodanes isolated from each sage species are described below. Particularly from Mexican salvias, a large number of clerodane derivatives with multiple substituents on the decalin ring and in the side-chain fragment have been isolated to date. Indeed, clerodanes comprise the majority of molecules identified from Mexican salvias, all belonging to the subgenus *Calosphace*. According to their chemical structure, these compounds isolated from Mexican salvias can be classified into the following ten subgroups: (1) clerodane-17,12–18,19-diolides (59 examples), (2) 15,16-epoxyclerodanes (38 examples) and (3) clerodane-18,19-diolida (30 examples), (4) C9-spiroclerodanes (28 examples), (5) seco-clerodane (23 examples), (6) 1,16-cycloclerodane (15 examples), (7) clerodanes-7,12-olides (14 examples), (8) 8,12-epoxyclerodan-18,19-olides (7 examples), (9) glycosylated clerodane (16 examples) and other clerodane derivatives (48 examples).

**Figure 11:**
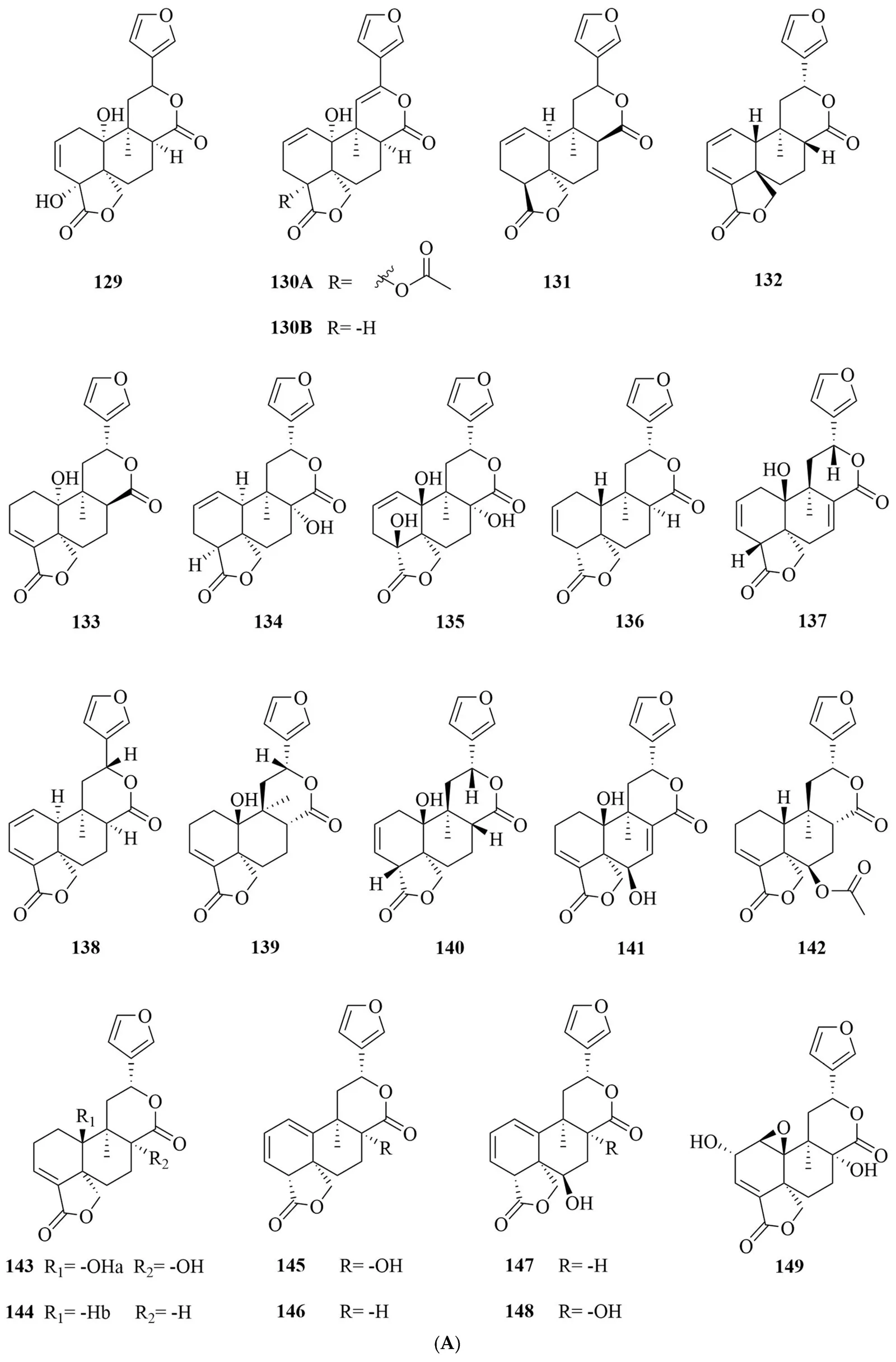

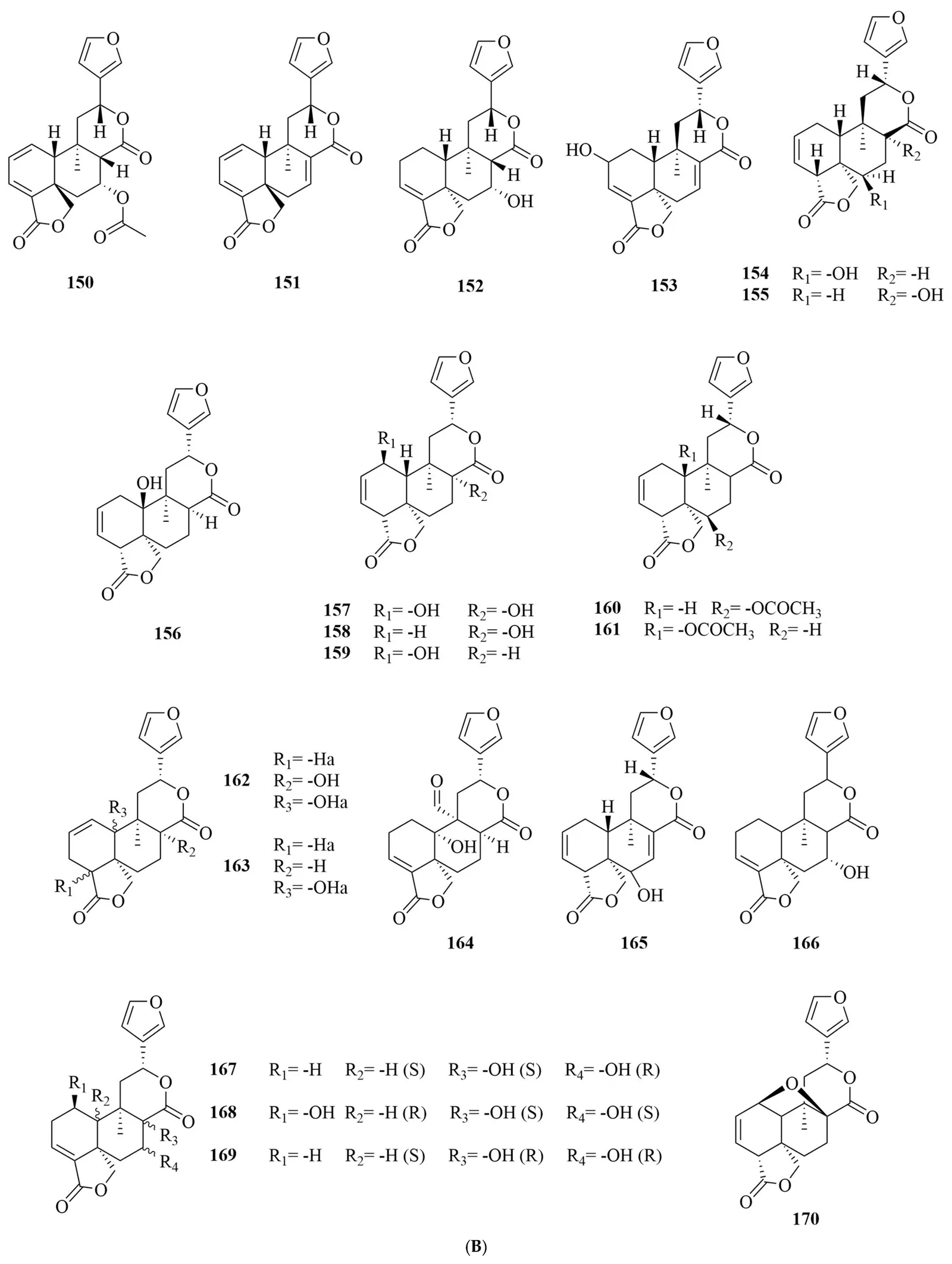

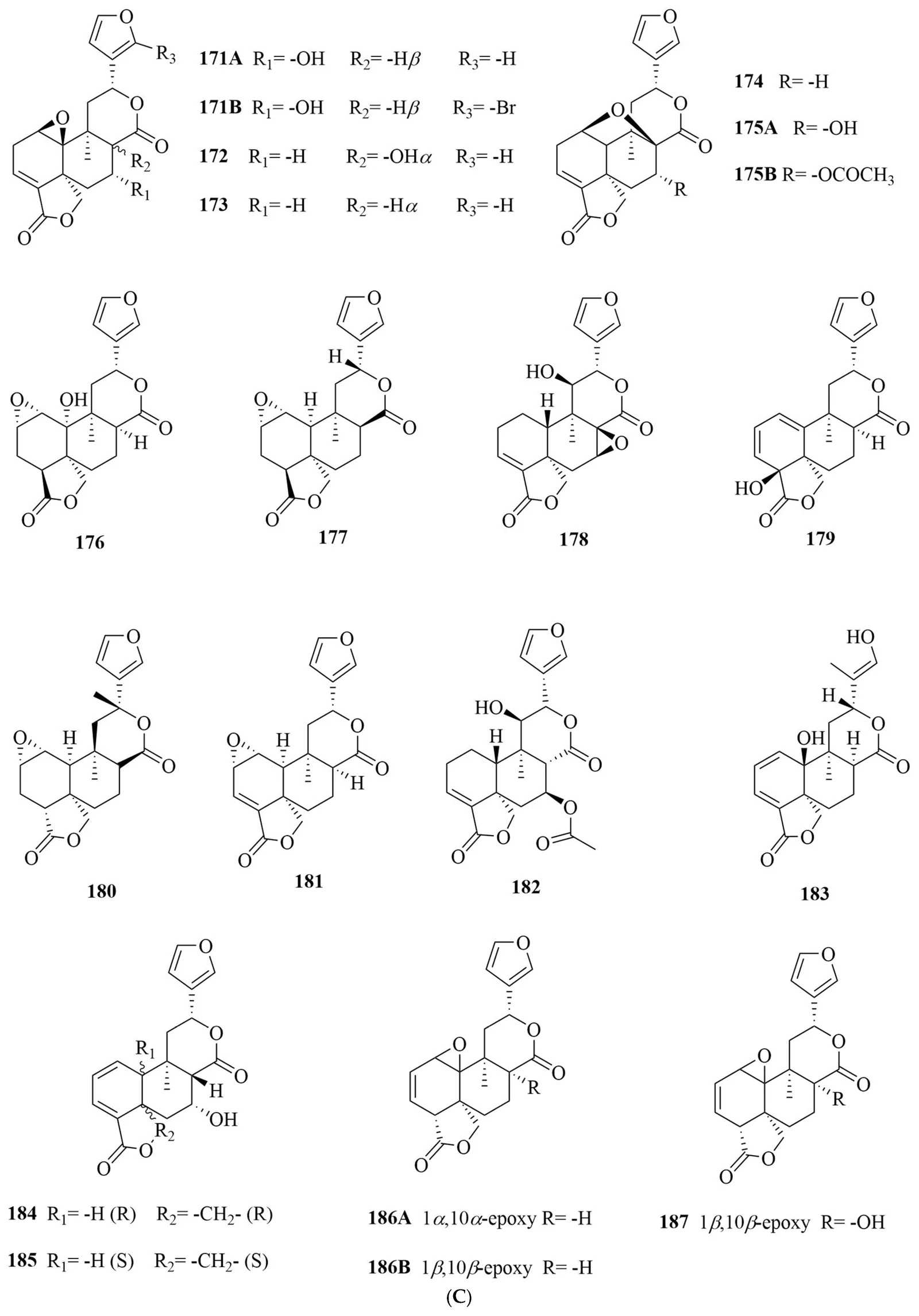
(**A**). Clerodane-17,12;18,19-diolides identified in Mexican salvias. (**B**). Continued. Clerodane-17,12;18,19-diolides identified in Mexican salvias. (**C**). Continued. Clerodane-17,12;18,19-diolides identified in Mexican salvias.

**Clerodane-17**,**12;18**,**19-diolides**. The chemical structures of the 59 reported clerodanes of this subgroup are shown in Figure 11 (structures **129**–**187**). All these diterpenoids have a very similar molecular structure with a furan ring inserted at carbon atom C12 and an α-lactone (oxolan-2-one) ring fused with the decalin moiety at atoms C4 and C5. An α-lactone (oxan-2-one) ring is fused through the atoms C9 and C8, except for compounds **129A-B** (4-O-acetylamarissinin derivatives), which contain a double bond in the ring δ-lactone [12]. A focused analysis of the type of substituents on the decalin ring reveals that at least one hydroxyl -OH group is bonded to one of the following carbon atoms: C2, C4, C6, C8, or C10 in 36 structures (~61%). Structures **129**, **141**, **143**, **149** and **157** have two hydroxyl groups and infuscatin (**135**) contains an uncommon tri-hydroxylated decalin ring at C4, C8 and C10 isolated from *Salvia shannoni* [97]. Other substituent groups present are acetyl (structures **130**, **142**, **150**, **175**, **182**, **186** and **187**), C1, C10-epoxy ring (structures **149**, **171**–**175**), C1, C2-epoxy ring (structures **176**, **177**, **180**, **181**) C1, C8-epoxy ring (structure **170**) and the formyl group (structure **164**). The only halogenated clerodane in this subgroup corresponds to the 16-bromotehuanine F structure (**168b**) from *Salvia herbacea* ([95]. Fifty-nine such structures have been identified in the subgenus *Calosphace* of the genus *Salvia*, in 26 species belonging to 14 sections. The sections with the highest number of species with this type of structure are *Holwaya* (*S. involucrata* Cav., *S. karwinskii* Benth. and *S. wagneriana* Pol.) [59,95,101] *Fulgentes* (*S. fulgens* Cav., *S. lineata* Benth., *S. microphylla* Kunth) [93] and *Polystachya* (*S. decora* Epling, *S. polystachia* Cav. (Syn. *S. filipes*) and *Salvia purepecha* Bedolla Lara et Zamudio [102,103,104]. The most widely distributed compound is salviarin, which has been recorded in *S. buchananii*, *S. carnea*, *S. gregii*, *S. karwinskii*, *S. reflexa*, and *S. rhyacophila* [50,93,105,106,107]. However, there are 47 structures found in only one species, such as amarissinin C and D in *S. amarissima* [12,98] and sepulturin C and D in *S. shannonii* [97] (Figure 11).

**Figure 12:**
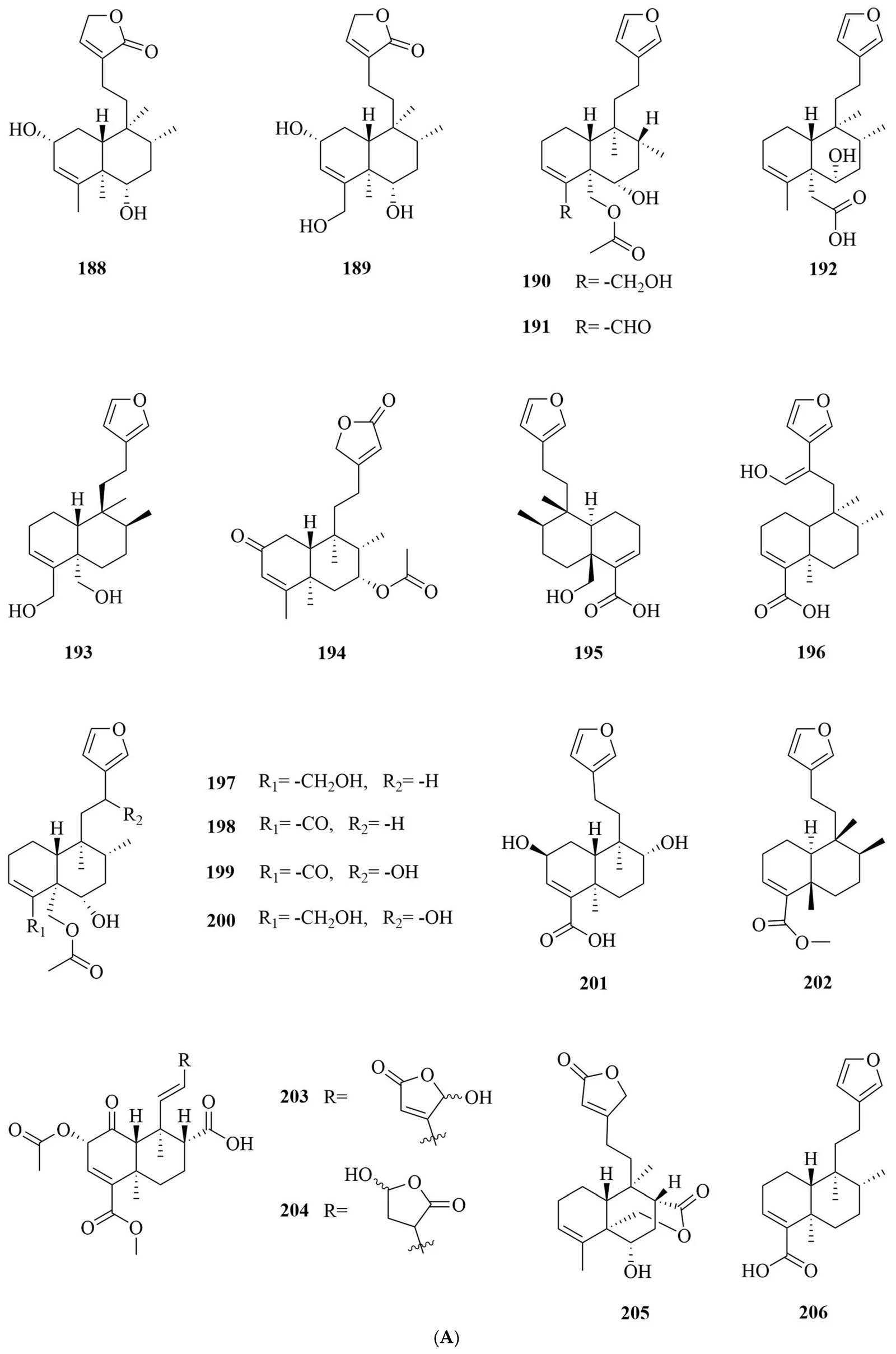

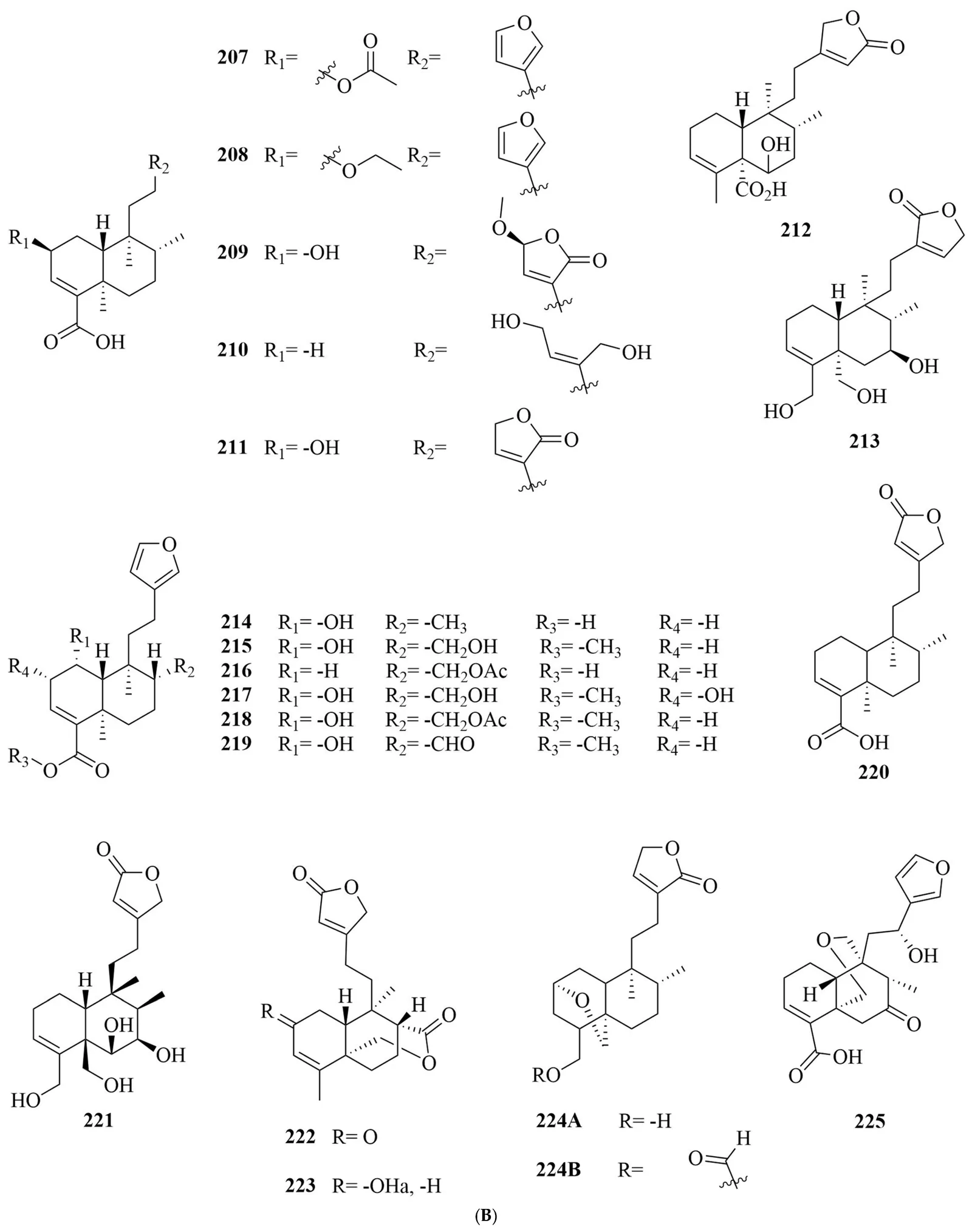
(**A**). 15,16-epoxyclerodanes identified in Mexican salvias. (**B**)**.** Continued. 15,16-epoxyclerodanes identified in Mexican salvias.

**15**,**16-Epoxyclerodanes**. The molecular structures of clerodanes **188**–**225** are shown in Figure 12. One of the main structural similarities between these molecules is a double bond in the decalin ring between the C3 and C4 atoms, which is present in 35 structures [8,90,93,101,108,109,110]. Only three molecules (hautriwaic acid, **195**; brevifloralactone, **224A** and **224B**) do not contain this double bond [101,111,112,113]. In addition, a *β*-unsaturated furan ring or an *α*,*β*-unsaturated lactone ring is attached at C12, except in **210** [93]**.** The decalin ring is commonly substituted with -OH groups on one of the following carbon atoms: C2, C4, C5, C6, or C8, except in 13 structures corresponding to hautriwaic acid (**195**), 12-hydroxyhardwickiic acid (**196**), hardwiickic acid methyl ester (**202**), salvidivin C (**203**), salvidivin D (**204**), hardwickiic acid derivatives (structures **206**–**208**, **210**), divinatorin C (**216**), clerodermic acid (**220**), brevifloralactone (**224**) and hispanin C (**225**) [33,93,97,114,115,116]. In several examples an acetyl group (structures: **190**, **191**, **194**, **197**–**200**, **203** and **204**) [8,19,49,93] or a carboxylic acid (structures **192**, **195**, **196**, **201**, **206**–**212**, **220** and **225**) [87,93,110] is present in the decalin ring predominantly at C4, C5, or C6. Only one structure includes a formyl substituent (**191**), obtained from *Salvia fulgens* Cav. [110]. The structures of salvimadrensin (**205**), brevifloralactone (**224**), and hispanin C (**225**) contain a lactone unit on the decalin ring [89,108,111]. In the subgenus *Calosphace*, 38 structures of this type have been isolated from 17 species belonging to 12 sections of which the best represented are *Scorodonia* (*Salvia breviflora* Moc. et Sessé ex Benth., *S. keerlii* Benth., and *S. melissodora* Lag. (Syn. *S. dugesii*) [21,111,112], as well as *Holwaya* (*S. adenophora* Fernald, *S. guevarae* Bedolla and Zamudio, and *S. involucrata* Cav.) [93,113]. In this group of clerodanes, only two structures are shared with more than one species; the rest have been isolated from a particular species. *S. divinorum* (Section *Dusenostachys*) is particularly important, as eight distinct, species-specific structures have been reported as significant due to their hallucinogenic properties and medicinal potential [8,87].

**Figure 13:**
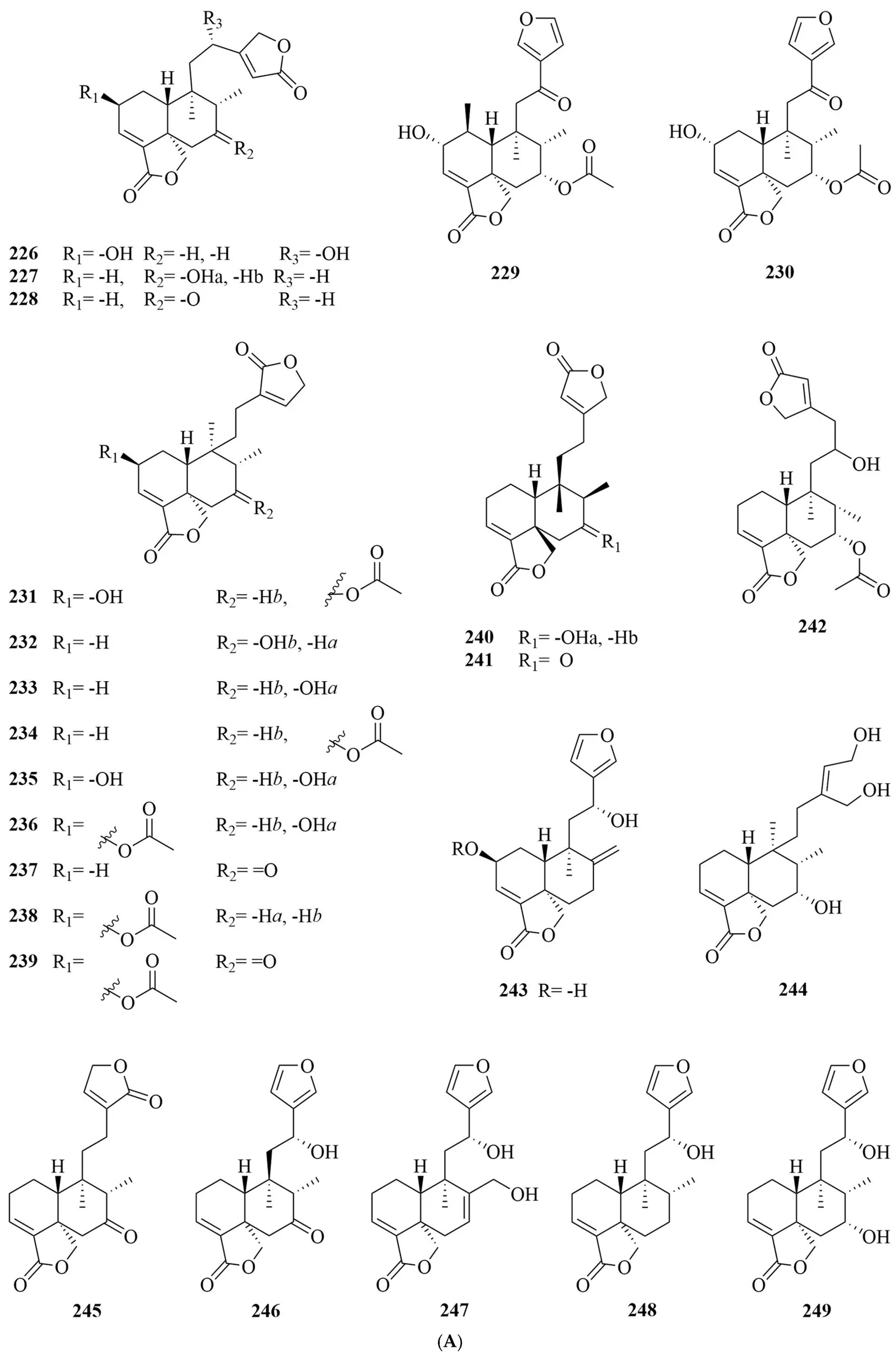

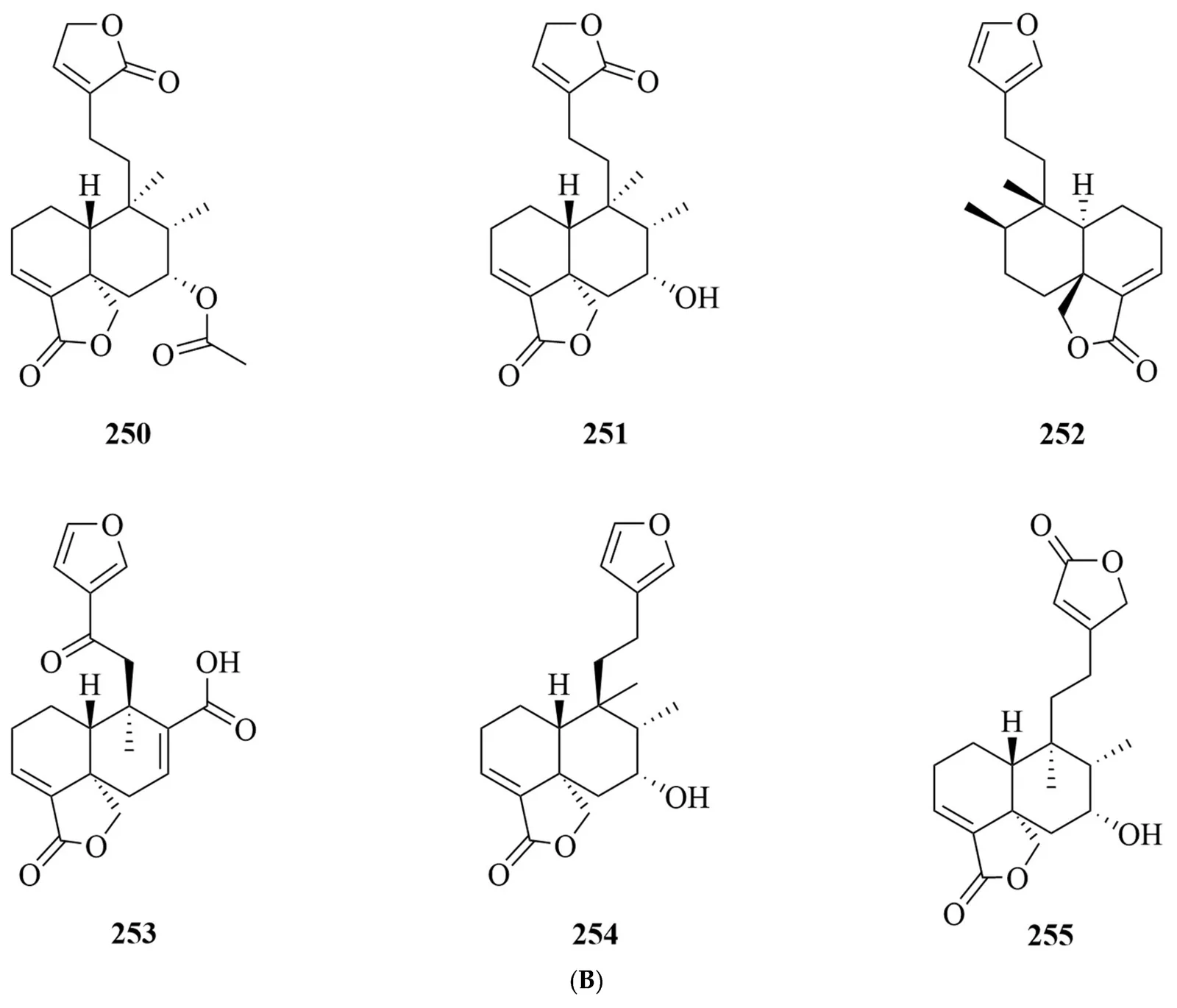
(**A**). Clerodane-18,19-diolide identified in Mexican salvias. (**B**). Continued. Clerodane-18,19-diolide identified in Mexican salvias.

**Clerodane-18**,**19-diolide**. Figure 13 shows the chemical structures of **226**–**255**. Overall, these molecules contain a double bond between C4 and C5 of the decalin ring (except lasianthin, structure **252**) [76], as well as a furan ring at C12 or an α,β-unsaturated lactone ring also at C12 (except portulide C, structure **244**), isolated from *Salvia melissodora* with two hydroxyl groups at C15 and C16 and characterized by spectroscopic tools [21]. Regarding the substituent group analysis, almost all structures contain hydroxyl groups on one of the decalin ring’s carbons, C2 or C7. Furthermore, a carbonyl or hydroxyl group at C12 was found in eight structures (salvisousolide (**229**), **230**, kerlinolide (**242**), aglycone rhynchospermoside A (**246**), hispanin I (**247**), bacchotricuneatin A (**248**) and 7α, 12α-dihydroxyhautriwaic acid-19-lactone (**249**)) [11,82,88,93,114]. Also, acetyl derivatives of these clerodanes were described in structures **229**–**231**, **234**, **242**, and **250**, which were isolated from *S. albiflora*, *S. urolepis*, *S*. *xalapensis* [82], *S. kerlii* [114], and *S. guevarae.* From this group, 30 structures have been isolated in the subgenus *Calosphace*, present in 12 species belonging to eight sections. The sections with the greatest number of structures of this type are *Angulatae* (*S. albiflora* M. Martens et Galeotti, Syn. *S. languidula*, *S. longispicata* M. Martens et Galeotti, *S. urolepis* Fernald, and *S. xalapensis* Benth.) [82,115,116], and *Scorodonia* (*S. keerlii* Benth. and *S. melissodora* Lag., Syn. *S. dugesii*) [21,22,101,114]. Salvisousolide is the compound shared by the four species of the *Angulatae* section, and only three structures are shared by two species; the rest are characteristic of a single species. In the latter case, *S. melissodora* (Syn. *S. dugesii*) stands out, with 10 clerodanes of this type, nine exclusive and 1-deoxybacrispine, which it shares with *S. involucrata* (section *Holwaya*).

**Figure 14:**
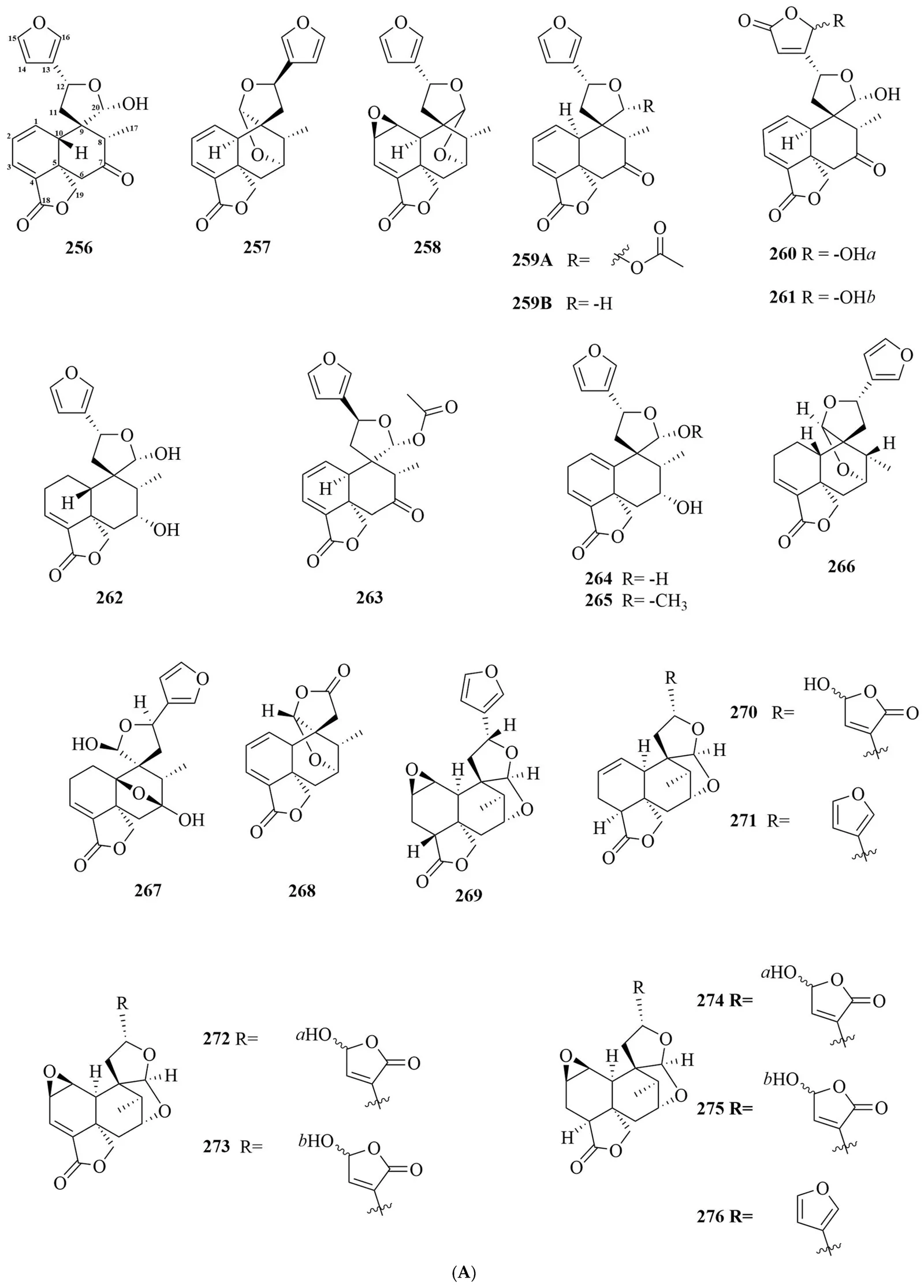

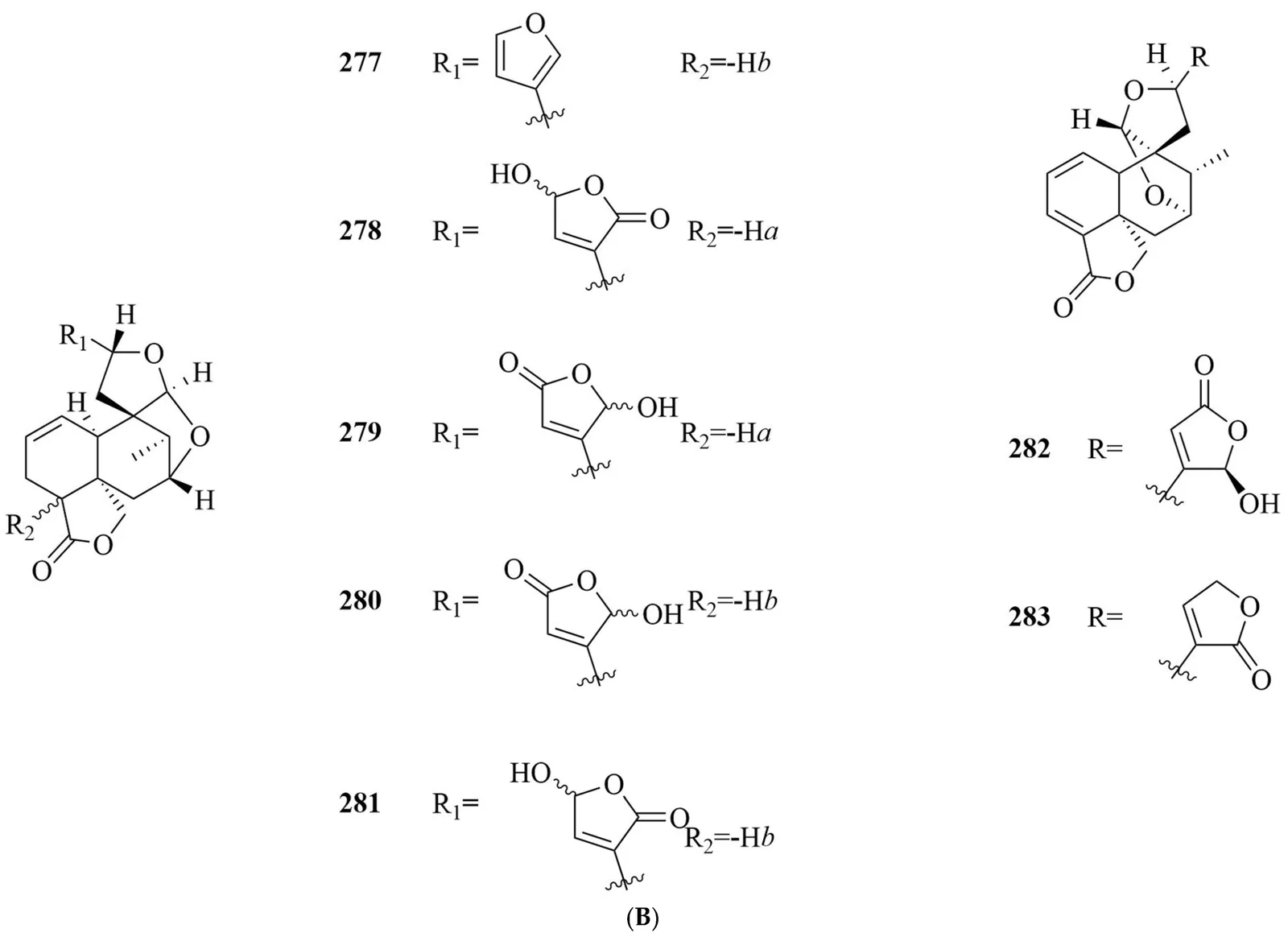
(**A**). C9-spiroclerodanes identified in Mexican salvias. (**B**). Continued. C9-spiroclerodanes identified in Mexican salvias.

**C9-spiroclerodanes**. Clerodane compounds in this group contain a C9-spirotetrahydrofuran substituted at C12 with a furan or butenolide ring (Figure 14). Some compounds exemplify a C20 variation connected to C7 by an oxygen atom (**257**, **258**, **266**, and **268**–**283**), forming two tetrahydrofuran rings joined at the C20 acetal. Other variations in the decalin ring, specifically compounds **258**, **269**, and **272**–**276**, contain a 1,2-oxirane group. Twenty-eight such structures have been reported in thirteen species of the subgenus *Calosphace*, located within nine sections. The sections with the highest number of compounds of this type are *Angulatae*, *Polystachya*, and *Scorodonia*, with two species each [29,91,96,102,117]. Six such clerodanes are found in more than one species, while 22 have been reported from a single species. Salvifaricin is the most widely distributed structure, as it is shared by nine species: *S. melissodora* (Syn. *S. dugesii*), *S. farinacea* Benth., *S. gesneriflora* Lindl. et Paxton, *S. hispanica* L., *S. leucantha* Cav., *S. polystachia* Cav. (Syn. *S. filipes*), *S. tiliifolia* Vahl., *S. tonalensis* Brandegee, and *S. urolepis* [11,89,100,118].

**Figure 15:**
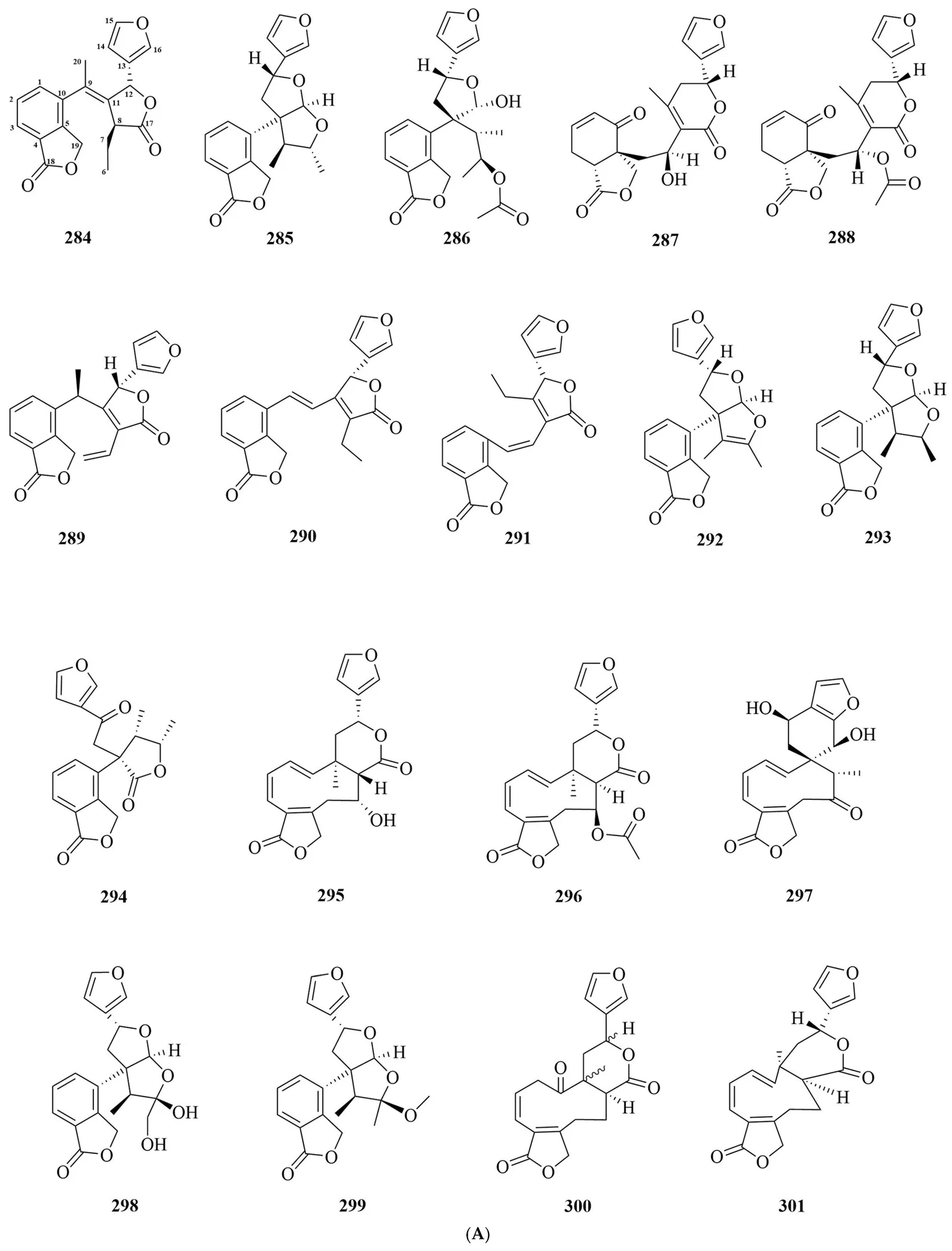

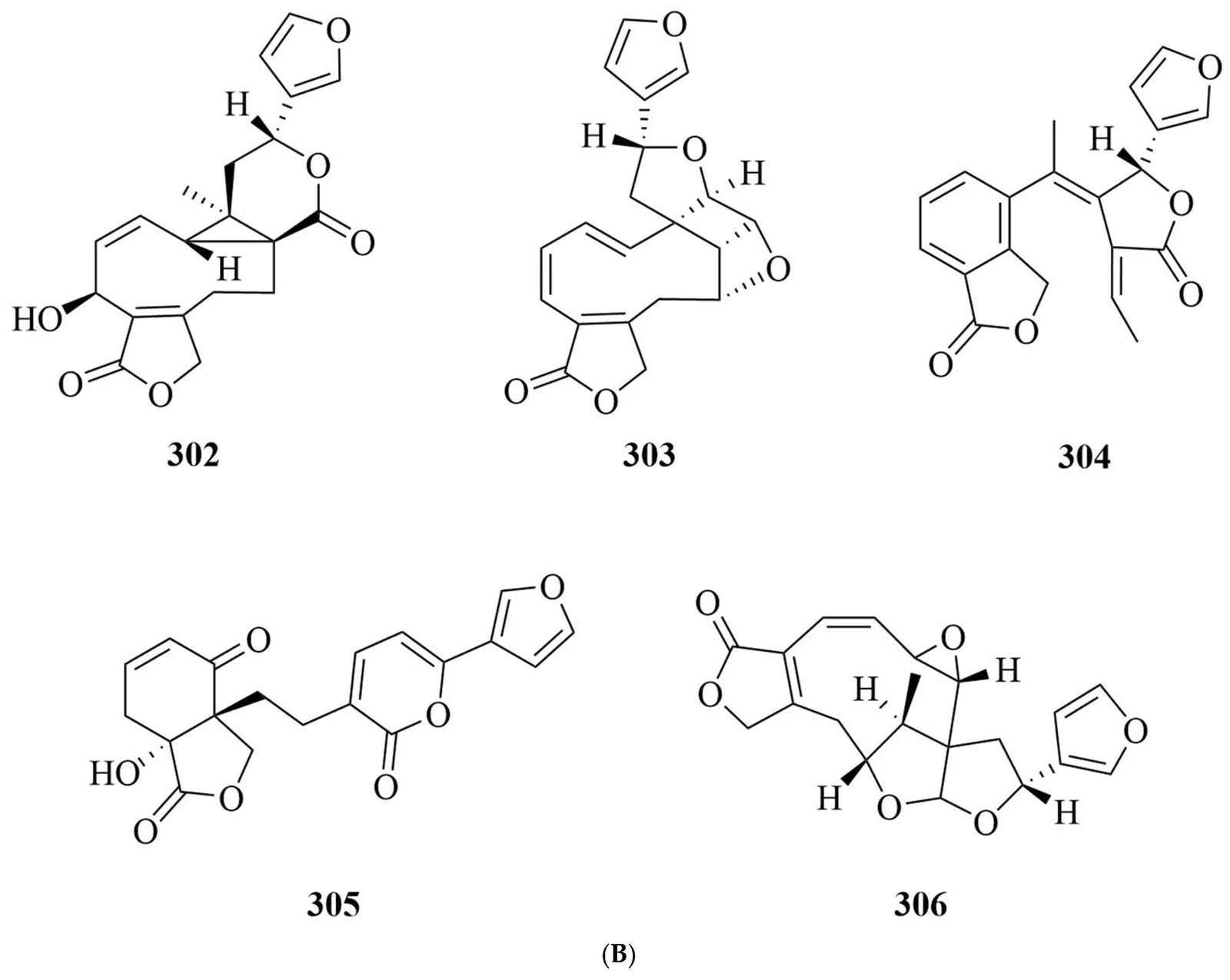
(**A**). *Seco*-clerodanes identified in Mexican salvias. (**B**). Continued. *Seco*-clerodanes identified in Mexican salvias.

**Seco-clerodanes***. Seco*-compounds have an open ring at some point in the clerodane skeleton, creating multiple structures with additional rearrangements (Figure 15). Examples include 5,6-*seco*-clerodanes, 9,10-*seco*-clerodanes and 5,10-*seco*-clerodanes. The 5,6-*seco*-clerodanes (**284**–**286**, **289**–**294**, **298**, **299**, and **304**) have novel skeletons characterized by cleavage of the C5/C6 bond and aromatization of the decalin A-ring. In contrast, the 9,10-*seco*-clerodanes (**287**, **288**, and **305**) contain a 17,12-δ-lactone. In the 5,10-*seco*-clerodanes (**295**–**297**, **300**, **301**, and **303**), the unsaturated patterns of the “opened” decalins include conjugated systems. Twenty-three such structures have been reported within the subgenus *Calosphace.* Nine sections have been found, the richest being *Angulatae*, with five species (*S. albiflora*, Syn. *S. languidula*), *S. rhyacophylla* (Fernald) Epling, *S. tiliifolia*, *S. uruapana* Epling, and *S. xalapensis* Benth [24,89,102,116,119,120,121]. Fourteen compounds are unique to one species; the most widely distributed *seco*-clerodane is tonalesin, which has been isolated from *S. farinacea*, *S. tonalensis*, and *S. uruapana* [11,121,122].

**Figure 16:**
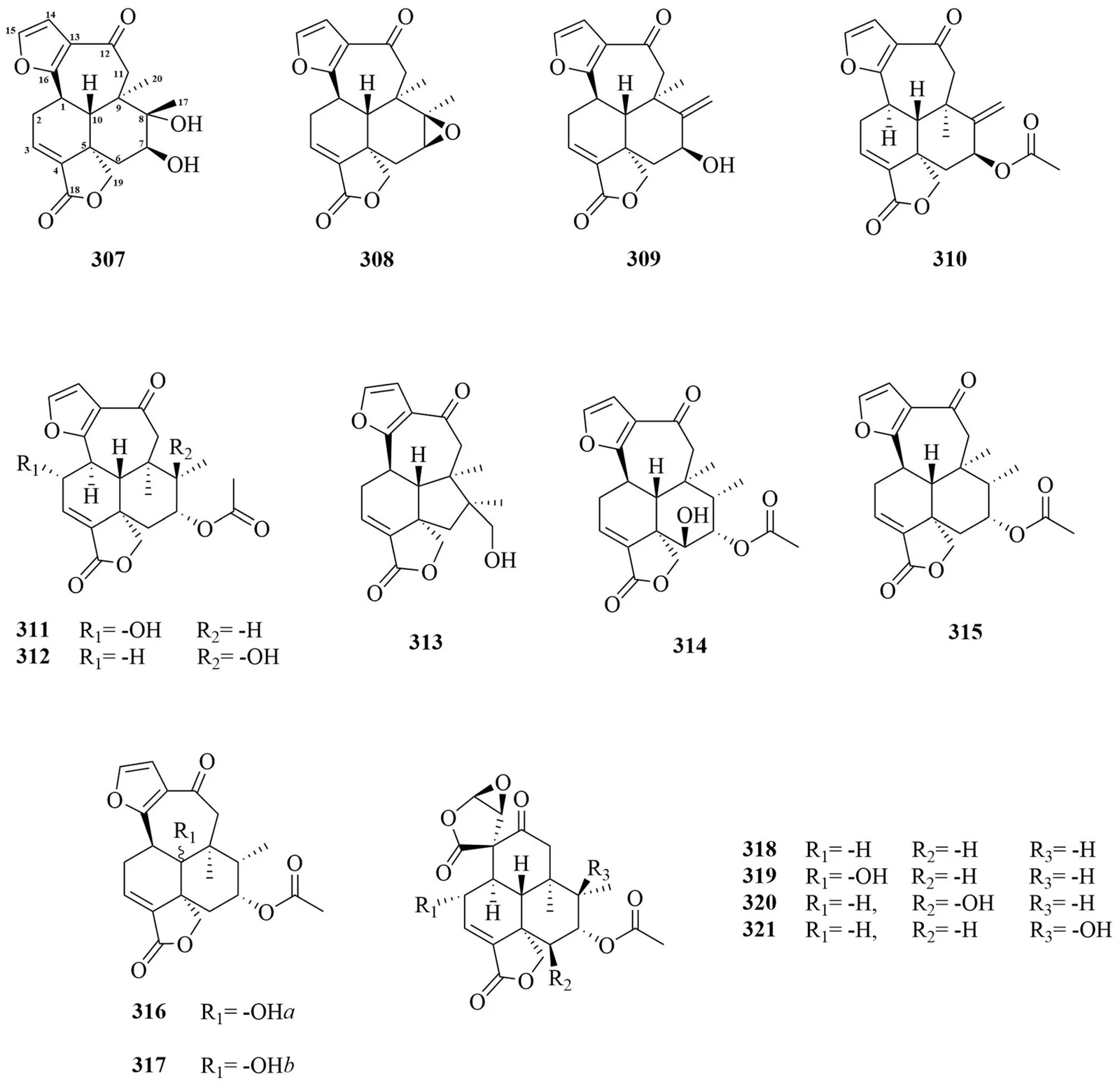
1,16-cycloclerodanes identified in Mexican salvias.

**1**,**16-cycloclerodanes**. The 1,16-cycloclerodanes (**307**–**317**) are rearrangement products of languidulane-type clerodane diterpenoids with a 6/5/7 or 6/6/7 tricyclic ring skeleton fused with a γ-lactone ring and a furan ring. On the other hand, 1,16-cycloclerodanes (**318**–**321**) are four diterpenoids with a rearranged clerodane skeleton containing a spiro γ-lactone epoxy function and a C1-C13 bond (Figure 16). There are 15 such structures in the subgenus *Calosphace*, across six species in six sections. All compounds are unique to one species, except salvixalapoxide, which is shared among *S. farinacea* and *S. xalapensis* [82,89].

**Figure 17:**
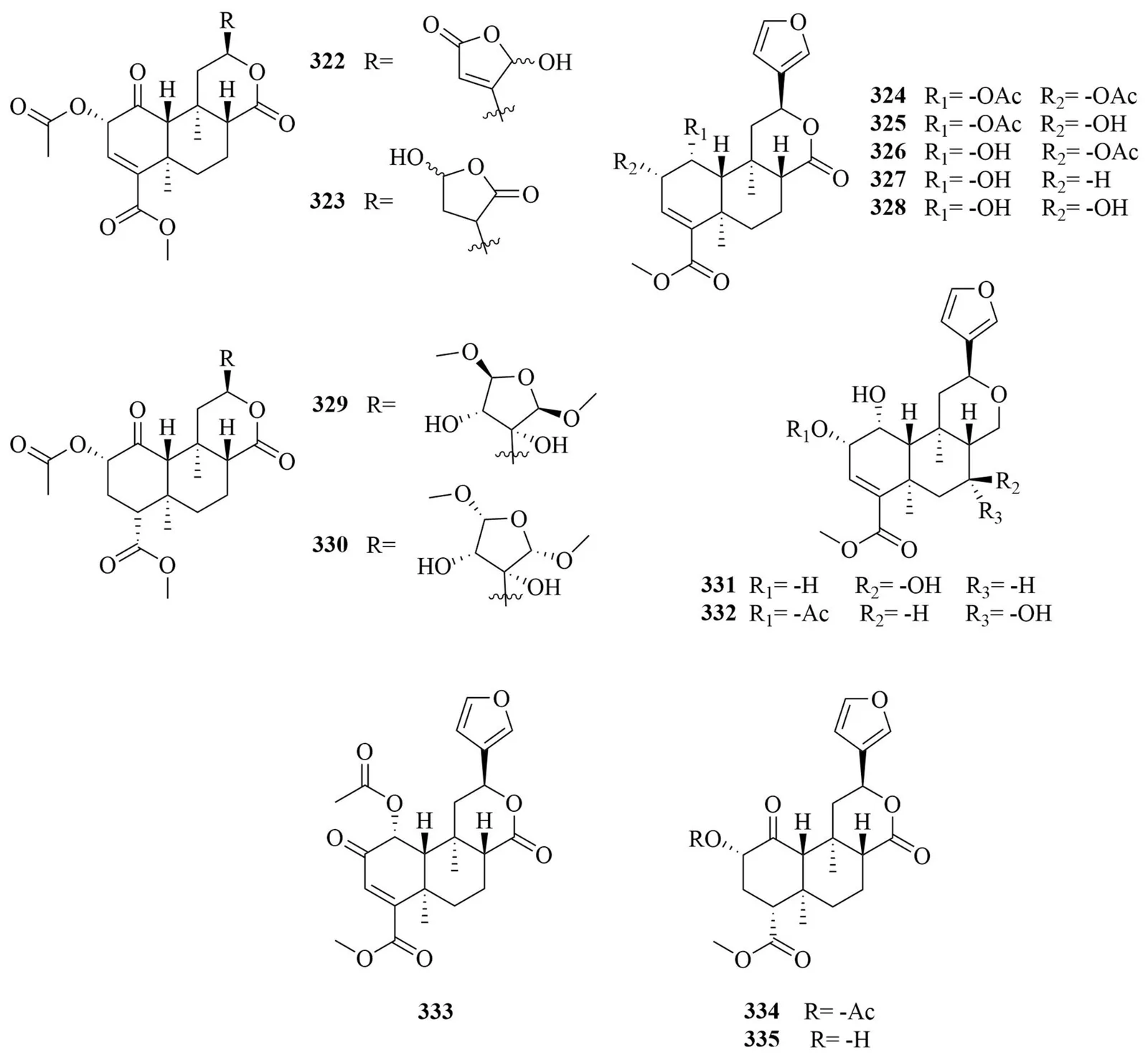
Clerodane-17,12-olides isolated from Mexican *Salvia* species.

**Clerodane-17**,**12-olides**. They are mainly characterized by a δ-lactone ring between C12 and C17 (Figure 17). In the case of structures **322**–**335** (salvinorin A-J, salvidivin A-B, salvinicin A-B), they also share a methyl ester at C18 and acetyloxy and hydroxyl groups at C1 and C2. Structures **331** (salvinorin I) and **332** (salvinorin J), the last salvinorins of the series isolated from *S. divinorum* to date, have a hydroxyl group at C7 [8,87]. On the other hand, **329** (salvicinin A) and **329** (salvinicin B) have a highly oxygenated tetrahydrofuran group at C12. Fourteen compounds of this type have been reported in the subgenus *Calosphace* and isolated from a single species, *S. divinorum* (section *Dusenostachys*) [8,87].

**Figure 18:**
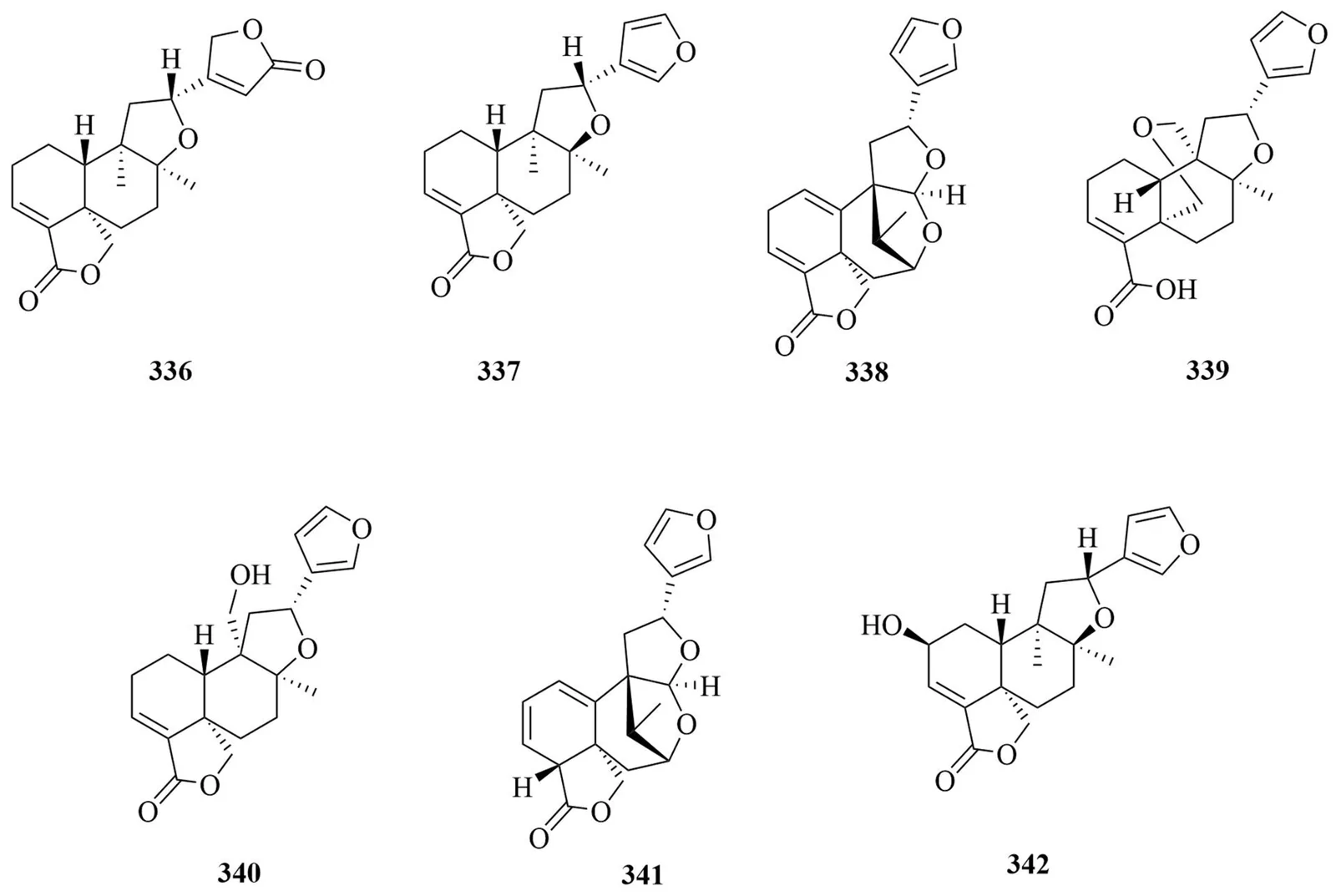
8,12-epoxyclerodanes-18,19-olides isolated from Mexican sages.

**8**,**12-epoxyclerodanes-18**,**19-olides**. The main similarity among these compounds is the epoxy ring between C8 and C12 (Figure 18). In the case of **336**–**342** isolated from Mexican sages, they present a ɣ-lactone between C18 and C19, with the singularity of **339** (hispanin B) that shows a reduction in the said ring. They also present a furan substituent at C12 (**337**–**342**), except for **336** (kerlin), which has a butenolide, or 2-furanone, substituent [88,90,123]. On the other hand, structures **338** (hispanin D) and **341** (hispanin E), instead of presenting an ordinary decalin ring, contain an eight-membered heterocycle with oxygen. It is important to highlight that this classification represents the lowest number among the wide variety of clerodane synthesized by Mexican sage species. Researchers have isolated seven structures of this type from six *Calosphace species* across six different sections. Dehydrokerlin is the most widely distributed compound since it is found in *S. fulgens*, *S. polystachia* (Syn. *S. filipes*), and *S. reptans* [100,110,124], the others are found in a single species. Finally, interestingly, *S. hispanica*, a species frequently used for food purposes, contains four clerodanes of this type.

**Figure 19:**
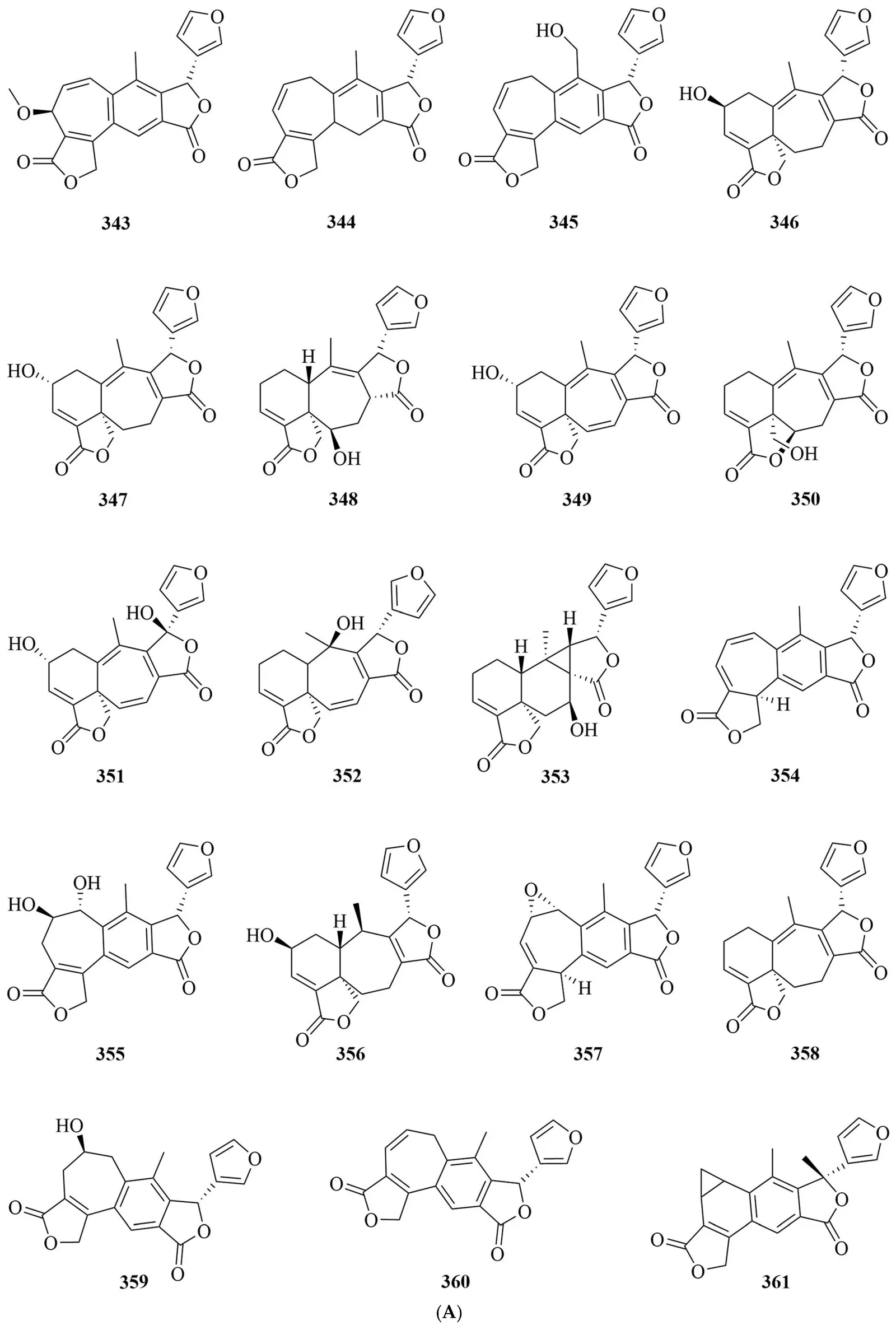

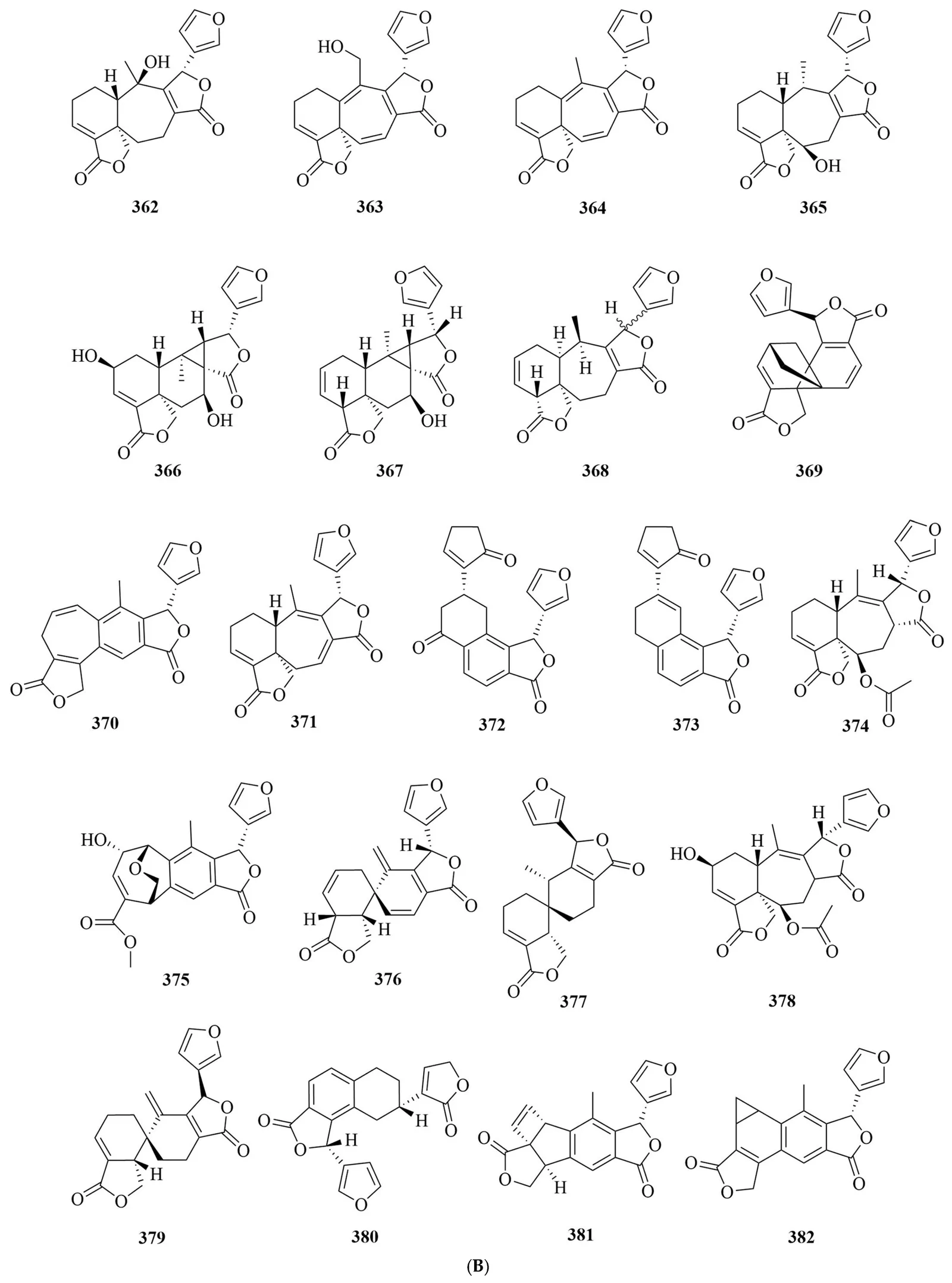

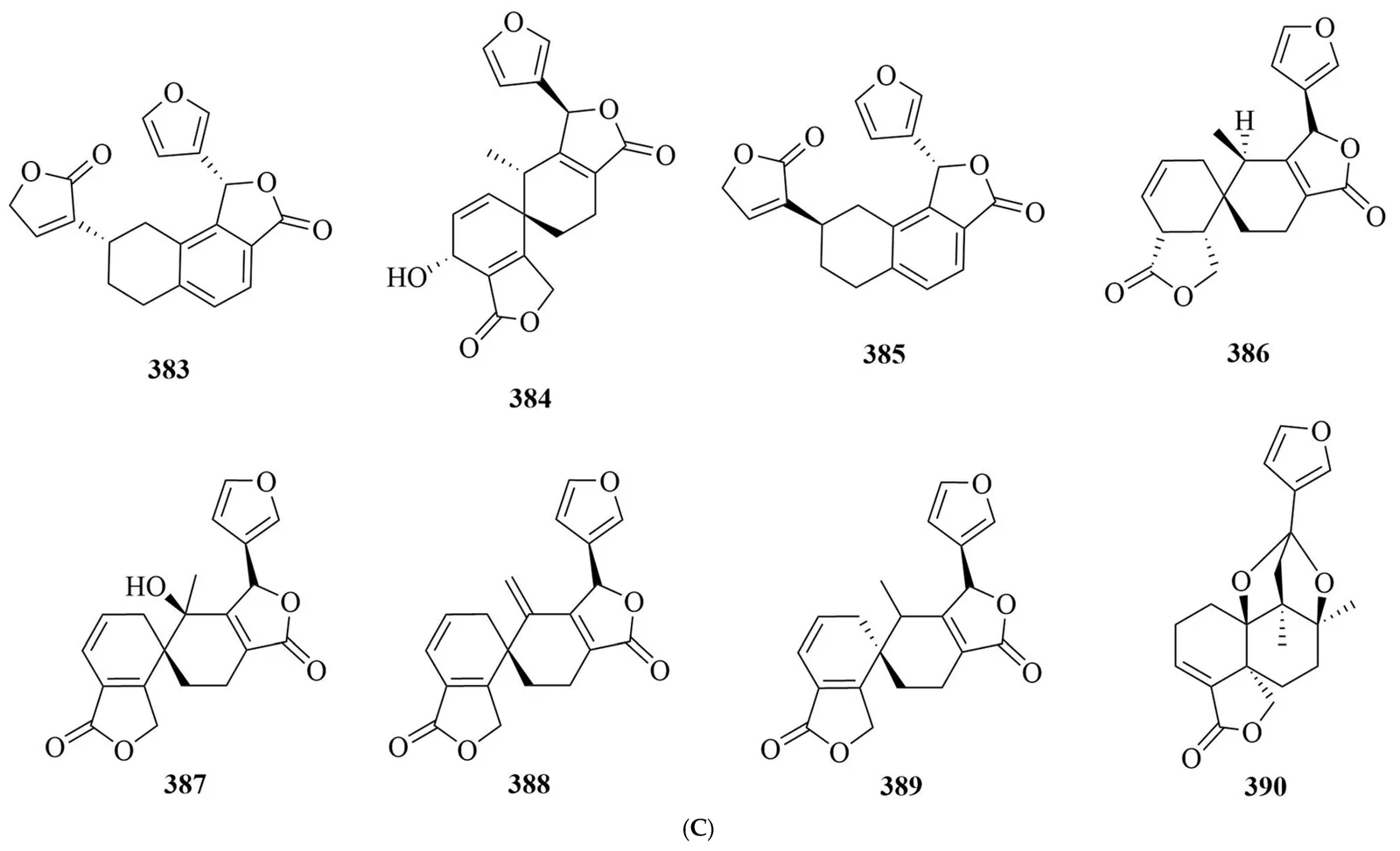
(**A**). Other clerodanes isolated from Mexican sages. (**B**). Continued. Other clerodanes isolated from Mexican sages. (**C**). Continued. Other clerodanes isolated from Mexican sages.

**Other clerodanes**. A total of 48 compounds isolated from Mexican *Salvia* species are classified in this section because they present unusual rearrangements of the clerodane skeleton. In *S. leucantha* and *S. hispanica*, 26 and 14 structures have been identified, respectively. Several of these structures (**343**–**352**, **354**–**360**, **362**–**365**, **368**, **370**–**371**, **374**–**377**, **378**) (Figure 19) contain a seven-membered cycle that can be biosynthesized via cyclization and oxidation from ordinary clerodane structures containing hydroxyl groups [26,28,29,32,82,91,94,101,114,115,120]. Of these compounds, **349** (salvianduline E), **360** (dugesin B), **370** (isosalvipuberulin/isopuberulin), and **374** (salvigenolide) are those that have been isolated from the most significant number of salvias, among which are *S. leucantha*, *S. dugesii*, *S. hispanica*, and *S. tiliifolia* [32,91,125]. Among these four compounds, salvianduline E stands out due to its hydroxyl group at C2. Another series of compounds in this sub-classification is distinguished for not presenting the decalin ring fused at C5 and C10, but only by one carbon (**376**–**377**, **379**, **384**, **386**–**389**) (Figure 19); most have been isolated from only one species of *Salvia*, where *S. leucantha* and *S. hispanica* stand out again [27,121,126,127]. This last class of compounds has been proposed to arise from oxidation-reduction and dehydration processes of precursors of the other clerodanes that contain seven-membered cycles. These clerodanes have been isolated from 19 species belonging to 16 sections, some distributed in more than three species such as tilifodiolide (**380**) in *S. albiflora* (Syn. *S. languidula*), *S. chamaedryoides*, *S. melissodora* (Syn. *S. dugesii*), *S. hispanica*, *S. leucantha*, *S. mexicana*, and *S. tiliifolia* [73,116,125,128], or salvianduline D from *S. blepharophylla*, *S. polystachia* (Syn. *S. filipes*), *S. lavanduloides*, *S. leucantha*, and *S. miniata* [65,102,129,130,131]. Within this classification of other clerodanes, structure **380** has been found in most *Salvia* species. It is mainly characterized by unsaturation in the B ring of the decalin system and, adjacent to C8 and C12, a ɣ-lactone linked to a furan group.

**Figure 20:**
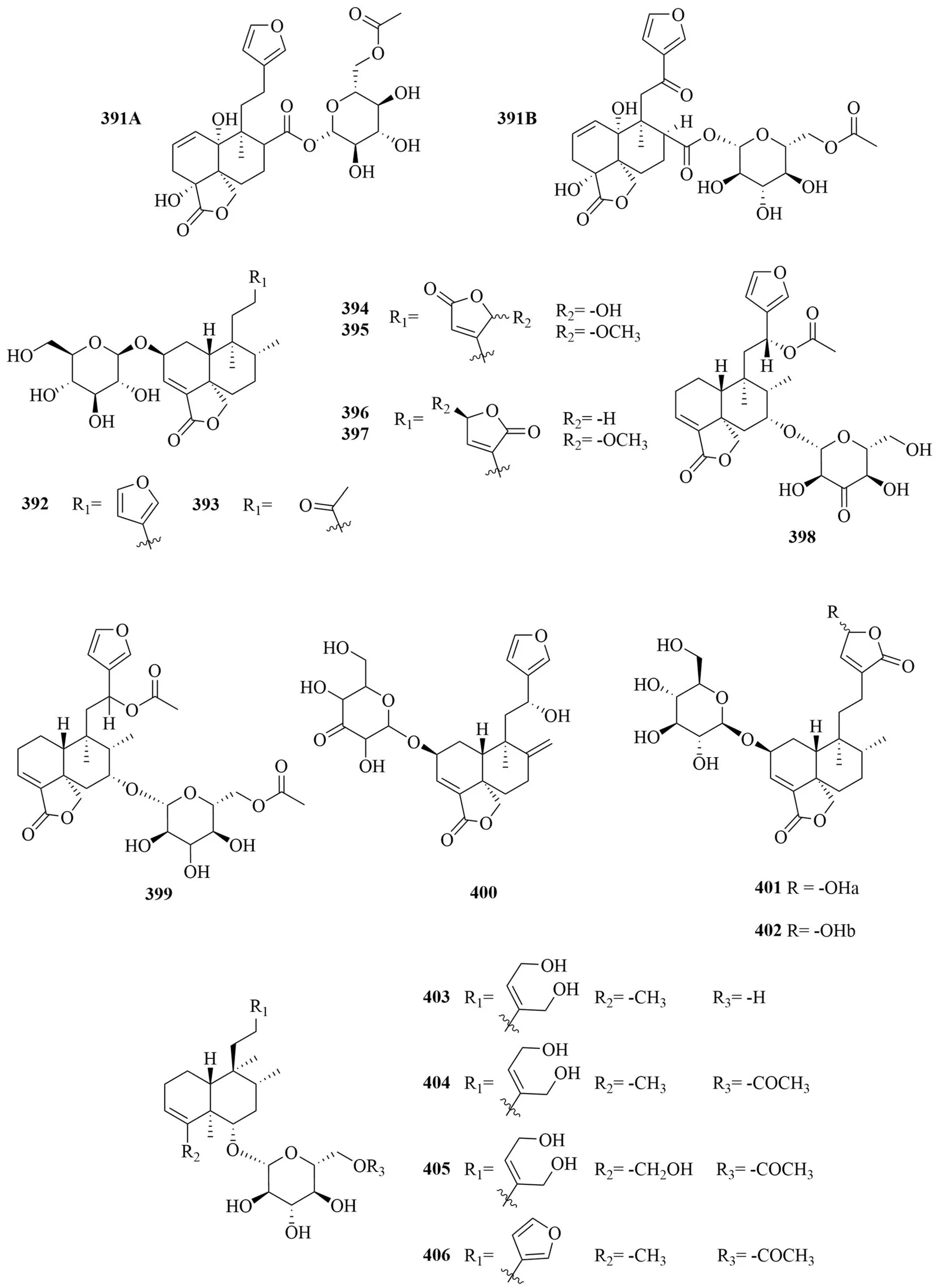
Glycosylated clerodanes isolated from Mexican salvias.

**Glycosylated clerodanes.** From the aerial parts of four species belonging to four sections, *S. amarissima*, *S. chamaedryoides*, *S. gregii*, and *S. hispanica*, 16 glycosylated clerodanes have been characterized. In their free form, i.e., without the glycoside molecule present, structures **391**–**402** belong to the clerodan-18,19-olides, structures **403**–**405** to clerodane-15,16-diols, and **406** to 15,16-epoxyclerodanes (Figure 20). In these structures, the glycoside is linked at C8 (**401**), C2 (**392**–**397** and **400**–**402**), C7 (**398**–**399**), and C6 (**403**–**406**). Structures **391**–**397** and **401**–**402** were isolated from *S. amarissima* and have in common the basic skeleton of a clerodan-18,19-olide and the glycoside linked at C2 or C8 [12,98,99,105]. The main difference between these molecules derived from *S. amarissima* is the presence of substituent groups on the oxidized furan derivative at C13. Structures **398** and **400** isolated from *S. chamaedryoides* and *S. hispanica*, respectively, have as a similarity a keto-glucopyranoside linked at C2 or C7; on the other hand, **391** from *S. amarissima* and **399** from *S. chamaedryoides* contain an acyl group in the glucoside [73,89]. Finally, compounds **403**–**406** isolated from *S. greggi* present a basic skeleton different from the rest of the glycosylated molecules identified in other species of Mexican sages, presenting a decalin ring without the presence of lactone rings but with two hydroxyl substituents, one at C15 and another at C16 (**403**–**405**), or a furan ring [105] (Figure 20).

### 3.6. Biological Activities

Compounds isolated from *Salvia* species exhibit a broad spectrum of biological activities. In this investigation, compounds isolated from *Salvia* subgenus *Calosphace* and/or *Audibertia* were identified as producing certain biological activities that were registered and organized in Table 1 and Table 2 according to the corresponding abietane or clerodane groups, respectively. The first column corresponds to the compound or compounds isolated from a specific species or species of *Salvia;* when more than one metabolite is reported, each was evaluated independently, not as a mixture.

Our research group previously developed a detailed characterization of the experimental models, potency, selectivity, and pharmacological evidence for these compounds in a publication dedicated to this topic [4]. In the present study, pharmacological information is presented solely as a general framework to facilitate discussion of the biological potential of the observed chemical profiles.

## 4. Evolution of Abietane- and Clerodane-Skeleton Diterpenes in Mexican Sages

### Distribution in Salvia Subgenus Calosphace

Among the two models tested, the evolution of the abietanes in the Neotropical sages can be better described by the ARD model, which showed better AICc scores (AICcw = 0.9559) than by the ER model (AICcw = 0.0440). On the other hand, the best fitted model for the evolution of the clerodanes was the ER model (AICcw = 0.6363 vs. AICcw = 0.3636 in the ARD model).

**Figure 21:**
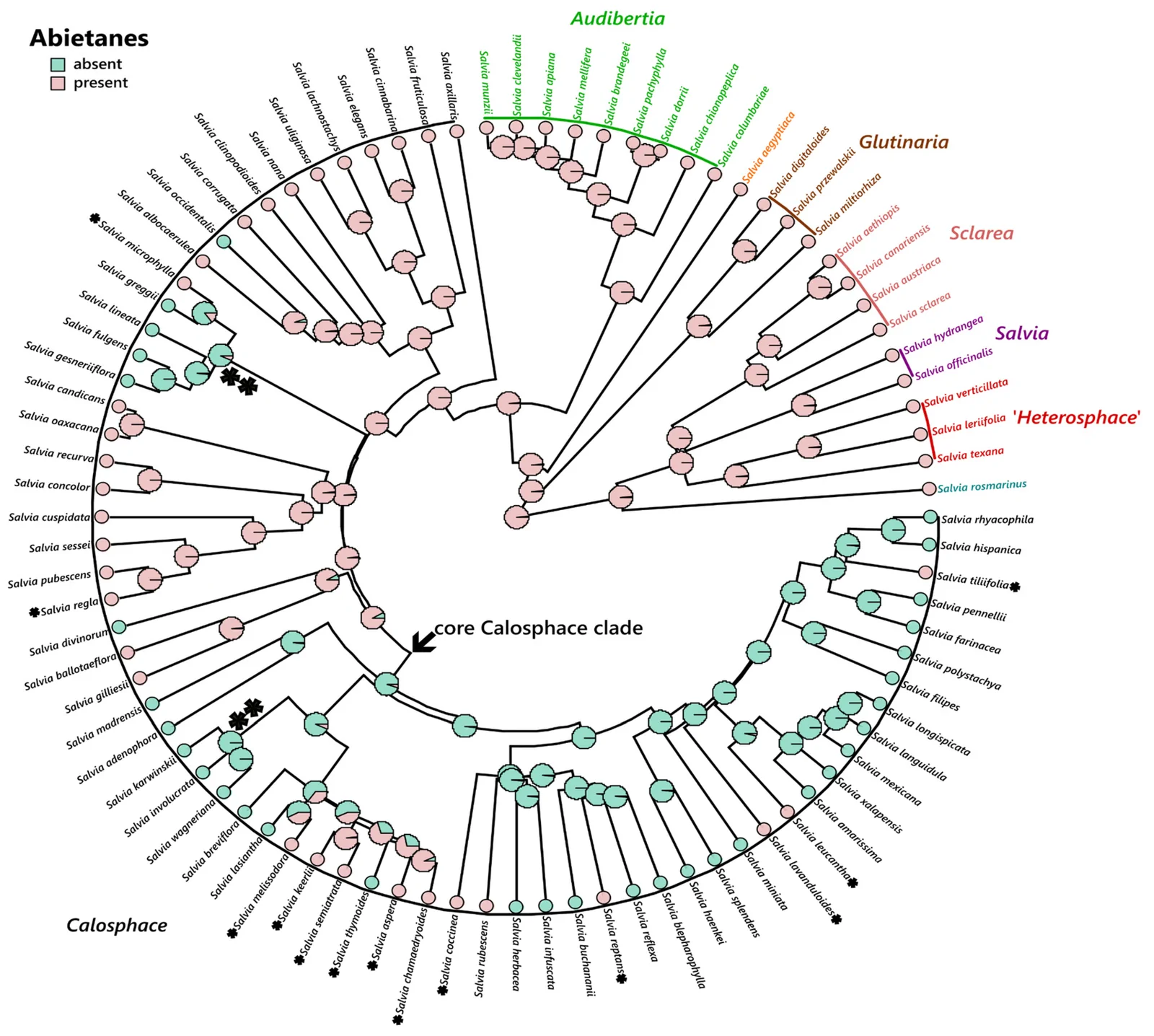
Ancestral state reconstruction using the likelihood method for abietanes diterpenoids in *Salvia* subgenus *Calosphace* and related lineages. Reconstructions were made under the ARD model. The *Salvia* s.l. lineages are color-coded. An asterisk (*) represents the species that produce both diterpenes, and two asterisks (**) represent the Fulgentes subclade, usually recovered as part of the core *Calosphace* clade in other phylogenies (i.e., refs. [6,13]).

**Figure 22:**
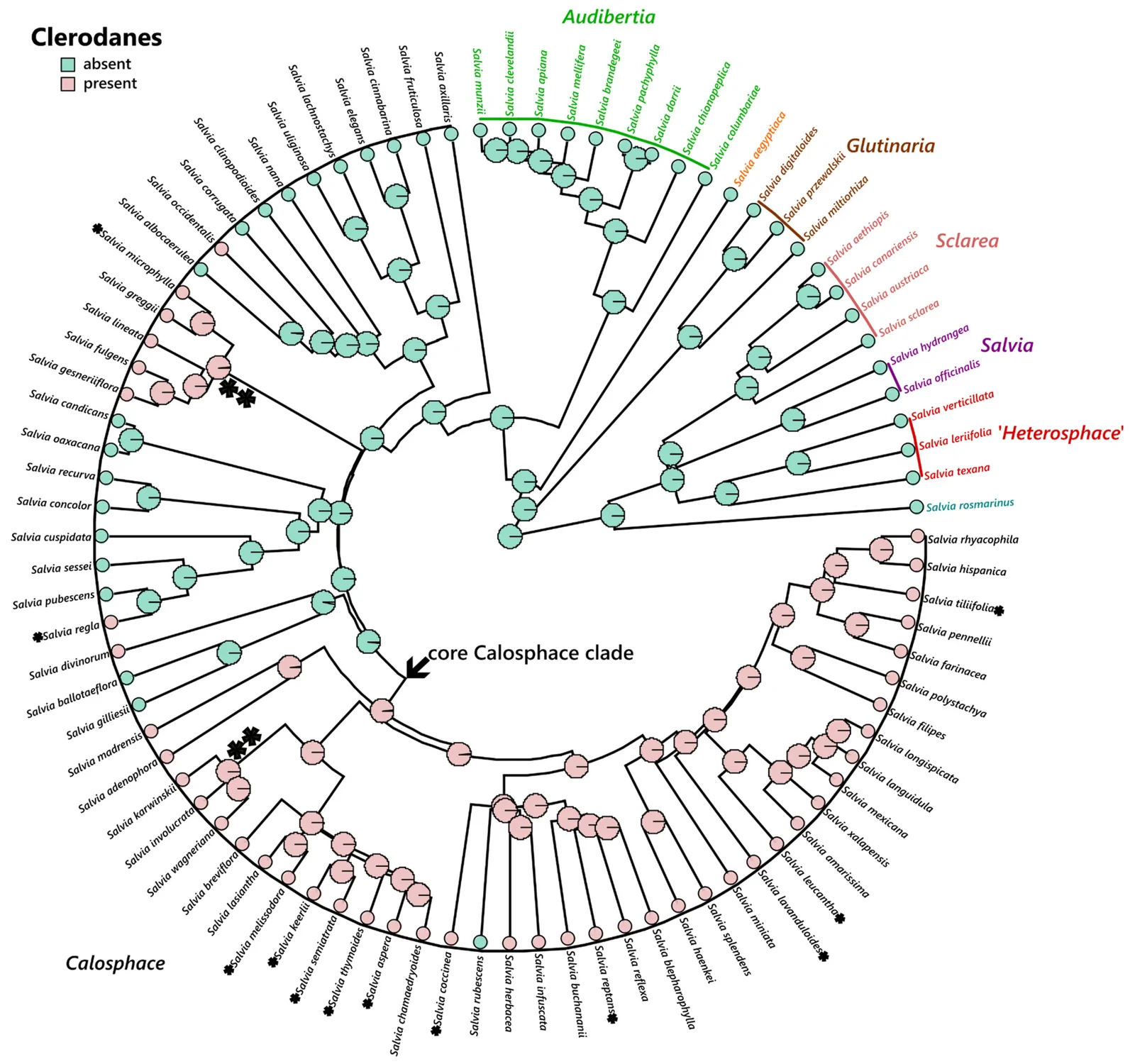
Ancestral state reconstruction using the likelihood method for clerodane diterpenoids in *Salvia* subgenus *Calosphace* and related lineages. Reconstruction was performed under the ER model. The *Salvia* s.l. lineages are color-coded. An asterisk (*) represents the species that produce both diterpenes, and two asterisks (**) represent the Fulgentes subclade, usually recovered as part of the core *Calosphace* clade in other phylogenies (i.e., refs. [6,13]).

Based on the information available so far, abietanes and clerodanes are almost differentially distributed in the lineages of *Salvia* (Figure 21 and Figure 22, respectively). Interestingly, many species that produce abietanes lack clerodanes and vice versa. However, there are a few species that synthesize both types of diterpenes, all of them belonging to *Salvia* subgenus *Calosphace*: *S. aspera*, *S. chamaedryoides*, *S. coccinea*, *S. keerlii*, *S. lavanduloides*, *S. leucantha*, *S. melissodora*, *S. microphylla*, *S. regla*, *S. reptans*, *S. semiatrata*, and *S. tiliifolia.* These species belong both to the early-diverging lineages of the subgenus and to the core *Calosphace* clade, which suggests independent gains of the metabolites, except for the taxa of sections *Atratae*, *Conzattiana*, *Flocculosae*, and *Scorodoniae* that form part of the same clade (*S. aspera*, *S. chamaedryoides*, *S. keerlii*, *S. melissodora*, and *S. semiatrata*). Thus, they share a more recent common ancestor with both diterpenes than with the other taxa.

The abietanes are mostly lacking in *Salvia* subgenus *Calosphace*, particularly in the species that form part of the most diverse group: the core *Calosphace* clade (Figure 21). The ancestral state reconstruction for the abietanes suggests that the common ancestor of *Salvia* s.l. produced this type of terpenoids and, later, they were lost in a fraction of the Neotropical sages (the core *Calosphace* clade) and independently in some species of subgenus *Audibertia* and “*Heterosphace*” (Figure 21).

Clerodanes are exclusively present, or at least in a greater concentration, in members of the Neotropical sages, mainly those in the core *Calosphace* clade (Figure 22). The reconstruction of this character shows a higher likelihood of the absence of clerodanes in the ancestor of *Salvia*. It also suggests a high likelihood of the origin of these diterpenoids in the core *Calosphace* clade, with independent gains in a few species belonging to the early diverging lineages of *Calosphace* (Figure 22).

To date, information on the diterpenoids present is available for only ca. 12% of extant species of *Salvia* subgenus *Calosphace.* Although the distribution pattern of abietanes and clerodanes in the Neotropical sages uncovered here is a step forward in understanding the evolution of these metabolites in the subgenus, our results should be regarded as a preliminary hypothesis due to limited sampling. According to our findings, it is likely that the common ancestor of the *Salvia* s.l. lineage produced abietanes but not clerodanes. The latter could have originated later in the group’s evolutionary history, during the diversification of the Neotropical sages. However, to corroborate these findings, the sampling needs to be expanded by researching taxa from poorly represented clades (e.g., Angulatae clade, South American clade, etc.), diversity centers (e.g., the Andes, Antilles, eastern South America, etc.), or environments (e.g., moist forests, dry forests, etc.). Species that produce both diterpenoids are scarce, and many occur in dry environments such as xeric shrublands or dry oak forests. This pattern agrees with the drought-tolerance role of diterpenoids found in other members of *Salvia* s.l. [182] and should be further explored in the Neotropical sages. However, the lack of both diterpenoids in many different species found in dry environments also needs to be addressed.

## 5. Conclusions

The genus *Salvia* in Mexico has 310 species across 61 sections, including three subgenera: *Calosphace*, *Audibertia*, and *Heterosphace*. Despite being the most diverse genus in the country, only 78 species have undergone phytochemical studies, in which clerodane- and abietane-type compounds have been isolated. Abietanes are chemical structures present in both *Audibertia* and *Calosphace*. However, clerodanes seem to be exclusive to a fraction of subgenus *Calosphace*, the core Calosphace clade. This finding suggests that clerodanes likely evolved within the subgenus. Pharmacological studies of isolated, pure abietane and clerodane diterpenes from species of *Salvia* in the *Calosphace* and *Audibertia* subgenera have shown critical biological activities, including antioxidant, anti-inflammatory, cytotoxic, cardioprotective, neuroprotective, antibacterial, antifungal, antiparasitic, and antiviral effects, among others. However, they represent only a small portion of the total already characterized in these interesting subgenera as potential alternatives and sources of new therapeutic drugs.

## Figures and Tables

**Table 1:** Biological activities of abietanes isolated from *Salvia* subgenus *Calosphace* and *Audibertia*.

Compound	Species	Biological Activity	Reference
**Subgenus *Audibertia***			
Sageone	*S. apiana*	Antiviral, cytotoxic, ligand μ-opioid affinity.	[46,132,133]
16-hydroxycarnosic acid	*S. apiana*	Antibacterial and antifungal.	[134,135]
Tanshinone-IIA	*S. brandegeei*	Cytoprotective, anti-inflammatory, antioxidant, anticoagulant, antithrombotic, neuroprotective, against respiratory diseases.	[136,137]
7α-acetoxyroyleanone	*S. brandegeei*	Cytotoxic and cytostatic.	[138,139,140]
Carnosol 16-hydroxycarnosol	*S. clevelandii*	Antiproliferative, cytotoxic, anti-inflammatory, antioxidant, cardioprotective, protective against kidney injury.	[45,141,142,143,144]
Ferruginol	*S. mellifera*	Antimicrobial, cytotoxic; antimalarial, anti-parasitic, neuroprotective.	[43,145,146,147]
Taxodione	*S. mellifera*	Cytotoxic; leishmanicidal, antimicrobial, antiprotozoal.	[146,148,149,150]
7α-hydroxyroyleanone	*S. mellifera*	Antioxidant.	[151]
**Subgenus *Calosphace***			
15-hydroxy-7-oxo-abieta-8,11,13-triene, sugiol, horminone, 8α, 9α-epoxy-7-ketoroyleanone, galdosol	*S. albocaerulea*, *S. aspera*, *S. coulteri*, *S. oaxacana*, *S. anastomosans*, *S. concolor*, *S. lavanduloides*, *S. reptans*, *S. semiatrata*, *S. chamaedryoides*, *S. mellifera*	Cytostatic, antiproliferative, antibacterial, and antioxidant.	[55,66,73,152,153,154,155]
Conacytone	*S. anastomosans*, *S. ballotiflora*, *S. candicans*, *S. pubescens*	Cancer quimiopreventive and anti-inflammatory.	[10,156]
Icetexone, 7,20-dihydroanastomosine	*S. anastomosans*, *S. carranzae*, *S. ballotiflora*, *S. candicans*, *S. pubescens*	Antioxidant, cytotoxic, antiproliferative, and immunomodulatory.	[10,80,157,158]
19-deoxyicetexone	*S. ballotiflora*, *S. carranzae*	Antidiarrheal, antispasmodic, antioxidant, anti-inflammatory, and antitumoral.	[78,80,157]
7α-acetoxy-6,7-dihydroicetexone	*S. ballotiflora*	Cytotoxic, anti-inflammatory, antiproliferative.	[10,80]
6,7,11,14-tetrahydro-7-oxo-icetexone	*S. ballotiflora*	Antioxidant and antiproliferative.	[10,80]
Cariocal, anastomosine; tilifolidione, 19-deoxyisoicetexone	*S. anastomosans*, *S. candicans*, *S. ballotiflora*, *S. semiatrata*, *S. thymoides*, *S. tiliifolia*	Cytotoxic, antiproliferative, and immunomodulatory.	[10,24,157,158,159]
Sessein, isosessein	*S. regla*, *S. sessei*	Antioxidant, anti-inflammatory, and antibacterial.	[160]
Clinopodiolide A, B, and C.	*S. clinopodioides*	Antiparasitic, antioxidant, and antidiarrheal.	[79]
Leucansalvialin J and G	*S. leucantha*	Neurothropic.	[32]

**Table 2:** Biological activities of clerodanes isolated from *Salvia* subgenus *Calosphace*.

Compound	Species	Biological Activity	Reference
Subgenus *Calosphace*			
(5R, 8R, 9S, 10R)-15,16-diol-15,16-dihydro-hardwickiic acid; (2S, 5R, 8R, 9S, 10R)-2-β-hydroxy-16-oxo-15, 16-dihydro-hardwickiic acid; 7α, 12α-dihydroxyhautriwaic acid-19-lactone; ent-15,16-epoxy-10,6-dihydroxycleroda-3,7,13(16), 14-tetraene-17,12;18,19-diolide; ent-19-O-acetoxy-15,16-epoxy-3,13(16), 14-clerodatrien-6,8,12-triol; Splendidin C; (5S,7R,8S,9R,10S,12R)-7,8-dihydroxycleroda-3,13(16),14-triene-17,12;18,19-diolide; (7R,8S,9R,12R)-7-hydroxy-5,10-seco-neo-cleroda-1(10),2,4,13-(16),14-pentaene-17,12;18,19-diolide; (5R,7R,8S,9R,10R,12R)-7-hydroxycleroda-1,3,13(16),14-tetraene-17,12;18,19-diolide; (5S,7R,8R,9R,10S,12R)-7,8-dihydroxycleroda-3,13(16),14-triene-17,12;18,19-diolide; ent-19-O-acetoxy-15,16-epoxy-3,13(16), 14-clerodatrien-6,18-diol; Salvigresin D	*S. adenophora*, *S. buchananii*, *S. chamaedryoides*, *S. fulgens*, *S. greggii*, *S. involucrata*	Antibacterial.	[73,93,105]
Teotihuacanin, amarissinin A-C, amarisolide F, involucratin A, kingidiol, salvileucalin B	*S. amarissima*, *S. involucrata*, *S. leucantha*	Cytotoxic, multidrug-resistance modulators, antiarthritic, anti-inflammatory and antirheumatic.	[12,27,101,161,162,163,164]
Amarisolide A	*S. amarissima*	Antidiabetic, antinociceptive, anti-inflammatory, antihyperalgesic, antiallodynic, and anticonvulsant.	[4,165,166,167,168,169]
Semiatrin, salviarin, dugesin E, isosalvipuberulin, 1(10)-dehydrosalviarin, kerlinolide, 6β-hydroxysalviarin, 7α-hydroxy-ent-clerodan-3,13-dien-18,19: 16,15-diolide	*S. aspera*, *S. semiatrata*; *S. buchananii*, *S. carnea*, *S. gregii, S. karwinskii*, *S. reflexa*, *S. rhyacophila*, *S. melissodora (Syn. S. dugesii)*, *S. carnea, S. leucantha*, *S. tiliifolia, S. involucrata*, *S. herbacea, S. lineata*, *S. wagneriana*, *S. kerlii*, *S. reptans*	Antimicrobial, antifeedant, and multidrug-resistance modulatory.	[94,170,171]
Sepulturin A, C, E, linearolactone, infuscatin, salvimicrophyllin D, microphyllandiolide, polystachyne E, Tehuanin D-F, H, 1α, 10α-epoxysalviarin	*S. buchananii*, *S. decora*, *S. shannonii*, *S. polystachia (Syn. S. filipes)*, *S. infuscata*, *S. microphylla*, *S. herbacea*, *S. lineata*	Cytotoxic, antiparasitic, antiprotozoal, antiamoebic, antigiardial, anti-inflammatory, antibacterial, antifungal, and phytotoxic.	[95,96,97,172,173,174,175,176]
Tilifodiolide	*S. chamaedryoides*, *S. melissodora (Syn. S. dugesii)*, *S. leucantha*, *S. mexicana*, *S. tiliifolia*	Antifeedant, antidiarrheal, vasorelaxant, anxiolytic, antidepressant, anti-inflammatory, and antinociceptive.	[128,170,177]
(1R,5S,7S,8S,9R,10R,12R)-1,7,8-trihydroxycleroda-3,13(16),14-triene-17,12;18,19-diolide	*S. chamaedryoides*	Antidiabetic and antibacterial.	[73]
7β-acetoxysalvimicrophyllin A	*S. decora*	Antidiabetic and inhibitory activity of hPTP1B.	[103]
Dugesin B	*S. melissodora (Syn. S. dugesii)*, *S. leucantha*, *S. mexicana*, *S. tiliifolia*	Acetyl cholinesterase-inhibitory.	[28]
Dugesin F	*S. melissodora (Syn. S. dugesii)*, *S. tiliifolia*	Activity against influenza virus.	[94]
Salvifarinin B	*S. farinacea*	Effect on reducing hepatic steatosis.	[89]
Salvifaricin	*S. hispanica*	Hypoglycemic activity.	[117]
Salvifiline A	*S. polystachia (Syn. S filipes)*	Cytotoxic, colagen (COL1A1) transcription inducer.	[96,102]
Tehuanin G	*S. herbacea, S. shannonii*	Anti-inflammatory and antiparasitic.	[95,97]
Salvianduline E	*S. melissodora (Syn. S. dugesii)*, *S. hispanica*, *S. lavanduloides*, *S. leucantha*, *S. tiliifolia*,	Antitrypanosomal.	[27]
Leucansalvialin G	*S. leucantha*	Neurotrophic.	[32]
Salvileucantholide	*S. leucantha*	Acetyl cholinesterase inhibitor.	[28]
polystachine G, 15-epi-polystachine G	*S. polystachia (Syn. S filipes)*	Cytotoxic, antibacterial, antifungal, and phytotoxic.	[96]
15-epi-salvifiline A	*S. polystachia (Syn. S filipes)*	Collagen transcription inducer.	[96]
7-keto-neoclerodan-3,13-dien-18,19:15,16-diolide	*S. semiatrata*	Antinociceptive and anxiolytic.	[178]
Salvinorin A	*S. divinorum*	Anxiolytic, antidepressant, antinociceptive, anti-inflammatory, neuroprotector and antiparasitic.	[8,173,179,180,181]

## Data Availability

All data generated or analyzed during this study are included in this published article and its Supplementary Information Files.

## Supplementary Materials

The following supporting information can be downloaded at https://www.mdpi.com/article/10.3390/molecules31183287/s1, Table S1: List of Mexican sage species, Table S2: List of abietane- and clerodane-type compounds isolated from Mexican sage species. It is important to note that the references for each of the 406 abietane- and clerodane-type compounds isolated from Mexican sage species are listed in Table S2 of the Supplementary Material. Some of these are cited in the main text, while references [183,184,185,186,187,188,189,190,191,192,193,194,195,196,197,198,199,200,201,202,203,204,205,206,207,208,209,210,211,212,213,214,215,216,217,218,219,220,221] are cited only in Table S2 of the Supplementary Material.
